# Engineering mono- and multi-valent inhibitors on a modular scaffold†
†Electronic supplementary information (ESI) available. See DOI: 10.1039/d0sc03175e


**DOI:** 10.1039/d0sc03175e

**Published:** 2020-12-17

**Authors:** Aurora Diamante, Piyush K. Chaturbedy, Pamela J. E. Rowling, Janet R. Kumita, Rohan S. Eapen, Stephen H. McLaughlin, Marc de la Roche, Albert Perez-Riba, Laura S. Itzhaki

**Affiliations:** a Department of Pharmacology , University of Cambridge , Tennis Court Road , Cambridge CB2 1PD , UK . Email: lsi10@cam.ac.uk ; Email: albert.perezriba@utoronto.ca; b MRC Laboratory of Molecular Biology , Francis Crick Avenue , Cambridge Biomedical Campus , Cambridge , CB2 0QH , UK; c Department of Biochemistry , University of Cambridge , Tennis Court Road , Cambridge CB2 1GA , UK

## Abstract

Here we exploit the simple, ultra-stable, modular architecture of consensus-designed tetratricopeptide repeat proteins (CTPRs) to create a platform capable of displaying both single as well as multiple functions and with diverse programmable geometrical arrangements by grafting non-helical short linear binding motifs (SLiMs) onto the loops between adjacent repeats. As proof of concept, we built synthetic CTPRs to bind and inhibit the human tankyrase proteins (hTNKS), which play a key role in Wnt signaling and are upregulated in cancer. A series of mono-valent and multi-valent hTNKS binders was assembled. To fully exploit the modular scaffold and to further diversify the multi-valent geometry, we engineered the binding modules with two different formats, one monomeric and the other trimeric. We show that the designed proteins are stable, correctly folded and capable of binding to and inhibiting the cellular activity of hTNKS leading to downregulation of the Wnt pathway. Multivalency in both the CTPR protein arrays and the hTNKS target results in the formation of large macromolecular assemblies, which can be visualized both *in vitro* and in the cell. When delivered into the cell by nanoparticle encapsulation, the multivalent CTPR proteins displayed exceptional activity. They are able to inhibit Wnt signaling where small molecule inhibitors have failed to date. Our results point to the tremendous potential of the CTPR platform to exploit a range of SLiMs and assemble synthetic binding molecules with built-in multivalent capabilities and precise, pre-programmed geometries.

## Abstract 2

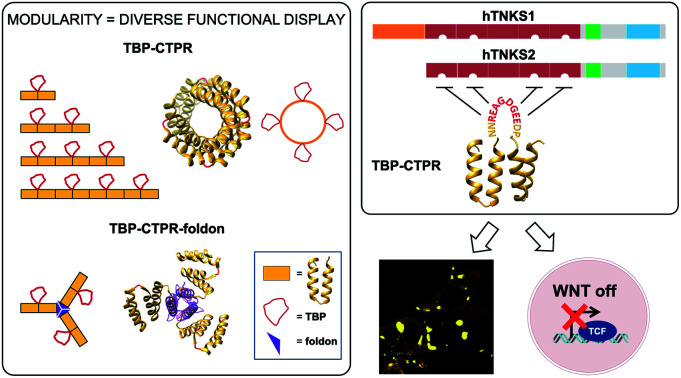
We exploit the simple modular architecture of repeat proteins to create a platform for single- and multi-functional display in diverse programmable geometries and demonstrate proof of concept by building potent inhibitors of a key signalling pathway.

## Introduction

The relationship between protein structure and function has been a cornerstone of biology for decades. However, in recent years, the unstructured or intrinsically disordered regions of the eukaryotic proteome (40% in humans) have gained increasing interest. This is due in part to the abundance in these regions of short independently functioning binding modules known as MoRFs (molecular recognition features) or SLiMs (short linear motifs).^1^ One approach to exploit these motifs for inhibiting protein–protein interactions (PPIs) is to chemically synthesise them in combination with modifications such as cross-linking and macrocyclisation designed to improve affinity, half-life and cell penetration.^2^ However, in nature high-affinity and high-specificity interactions and more complex regulatory mechanisms are achieved through multivalency and avidity, neither of which are straightforward to realise with conventional peptide technologies. It is also uncertain whether small molecules could ever act as effective inhibitors of such complex networks of multivalent contacts.^3^,^4^ Consequently, in order to interrogate and ultimately to drug such intricate networks of inter-linked motifs, new technologies are needed. There are many multivalent antibody technologies leveraging the natural modularity (*i.e.* multi-domain nature) of immunoglobulins.^5^,^6^ However, multivalency has been less successful in antibody-like domains, where it has only been achieved by connecting monovalent units in “beads-on-a-string” or by directly assembling them on antibody scaffolds.^7^–^10^ Likewise, functional peptide motifs have been assembled on synthetic chemical scaffolds,^11^,^12^ DNA^13^,^14^ and protein scaffolds,^15^–^21^ but no modular platform has been developed to date that is capable of combinatorial incorporation of multiple SLiMs in a single stable and reliable scaffold as well as presenting them with varied, precise and programmable geometries. Here we show that tandem-repeat proteins possess all the necessary features with which to build such a platform.

**Fig. 1:**
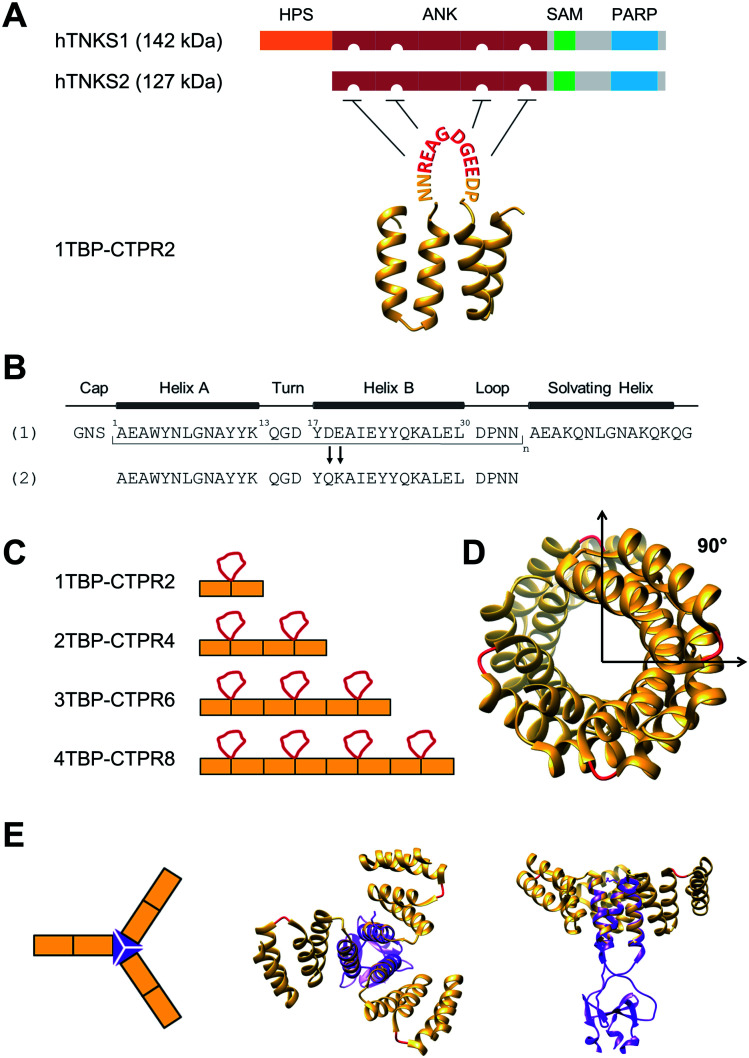
Design of hTNKS-binding CTPR proteins in different formats and valencies. (A) (Top) Domain architecture of hTNKS1 and hTNKS2, comprising a histidine, proline, serine-rich (HPS) domain, a substrate-binding ankyrin-repeat (ANK) domain made up of five ankyrin-repeat clusters (ARC), a sterile alpha motif (SAM) and the catalytic PARP domain. (Bottom) Schematic representation of 1TBP-CTPR2 construct, showing the hTNKS-binding peptide (TBP) grafted onto the loop between adjacent CTPR repeats (PDB 2HYZ) in order to inhibit hTNKS activity. As denoted by the semi-circular cut-outs, four of the five ARCs can bind to the TBP. (B) Sequence of the CTPR motif designed by Main and co-workers (1) and that used for this study (2).^31^,^63^,^64^ Secondary structural elements are shown. (C) Schematic representation of the *n*TBP-CTPR2*n* constructs. Each CTPR is shown as a yellow rectangle, and the TBP grafted onto the inter-repeat loops are in red. The amino-acid sequence of each construct is provided in Table S1.† (D) Crystal structure of CTPR8 (PDB ; 2HYZ) viewed along the superhelical axis. CTPR helices are in yellow and the inter-repeat loops, onto which the TBP sequence is grafted, are in red. (E) (Left) Schematic representation of CTPR2-foldon. Each CTPR is represented by a yellow rectangle and the foldon domain by a purple triangle. (Middle and Right) Different views of the modelled structure of CTPR2-foldon. The model was generated by grafting the foldon helix (PDB: ; 1OX3) onto the CTPR helix (PDB: ; 2HYZ) using the UCSF Chimera software 1.8.1.^65^ CTPR helices are shown in yellow, the inter-repeat loops, onto which the TBP is grafted, are in red.

Tandem-repeat proteins comprise tandem arrays of small structural motifs that pack in a linear fashion to produce regular, elongated, quasi-one-dimensional architectures and function in binding to other proteins, small molecules or nucleic acids. The repetitive modular organisation of this architecture makes it straightforward both to dissect and to redesign the biophysical properties.^22^–^27^ One of the simplest repeat structures is the tetratricopeptide repeat (TPR),^28^,^29^ a 34-residue motif comprising two antiparallel α-helices with adjacent repeats connected by a short turn (Fig. 1A, bottom).^30^ Artificial proteins comprising multiple copies of a consensus-designed TPR (CTPR) sequence have been shown to be extraordinarily stable.^31^ Moreover, the modular nature of the architecture means that consensus repeats are self-compatible and can be individually designed and put together in any order. We recently showed that the CTPR scaffold can accommodate peptide extensions in the loop between adjacent repeats up to as many as 25 amino acids without compromising the native structure.^32^,^33^ We then demonstrated that we could graft a SLiM from the protein Nrf2 that recognises the oncogenic protein Keap1 onto the scaffold and that we could not only recapitulate the native binding affinity but also improve it without need of sophisticated computational modelling. Thus, by combining this modular scaffold with SLiM grafting we potentially have the capacity for diverse functionalization.^33^

As proof of concept, we herein construct a set of monovalent and multivalent CTPR proteins to bind and inhibit the human poly (ADP) ribose polymerase (PARP) proteins tankyrase 1 and tankyrase 2 (referred to subsequently as hTNKS). hTNKS are unique in the PARP family in having a series of ankyrin-repeat cluster (ARC) subdomains (Fig. 1A).^34^,^35^ Four of the five ARCs (ARCI, ARCII, ARCIV and ARCV) mediate protein–protein interactions including substrate recognition by binding short (8 residue) peptide motifs.^36^–^39^ hTNKS has been implicated in the regulation of a number of cellular processes,^40^–^43^ but more recently the role of hTNKS in the regulation of Wnt pathway activity has received particular attention.^44^,^45^ The Wnt pathway is the principle driver of colon cancer, and a number of studies have reported the development of small molecule inhibitors of the hTNKS catalytic activity as a means of modulating Wnt pathway activity.^44^,^46^–^49^ However, by targeting the catalytic PARP domain these small molecules may lack specificity for hTNKS over other PARP family members.^50^–^52^ Moreover, they can only inhibit the catalytic function of hTNKS but not the well-documented non-catalytic functions.^53^,^54^ To overcome these limitations, our aim was to target the unique substrate-binding ARC subdomains and thereby block both catalytic and non-catalytic functions. Guettler *et al.* previously determined a consensus sequence that is recognised by the ARC subdomains (referred to subsequently as the tankyrase-binding peptide or TBP)^36^ (Fig. 1A), and we have shown that macrocyclised TBPs are able to bind hTNKS and inhibit Wnt signalling.^55^

We grafted the TBP onto the inter-repeat loop of the CTPR scaffold to generate a series of mono- and multi-valent binding proteins (*n*TBP-CTPR2*n*). Our aim was to explore the effects of multivalency on the biophysical properties of the proteins and on their interactions with and cellular inhibition of hTNKS, which is itself multivalent. Further motivation came from the fact that several hTNKS substrates are themselves multivalent as they contain multiple TBPs.^56^ In order to further extend the multivalent capabilities and to produce more complex binding geometries beyond what is possible with the linearly arrayed *n*TBP-CTPR2*n* format, we engineered a trimeric ‘foldon’ motif into the constructs (*n*TBP-CTPR2*n*-foldon).^57^–^61^ Our results highlight the power of multivalent inhibitors for targeting complex protein–protein interactions, where occupancy-driven inhibition using simple monovalent molecules is unlikely to be effective.

## Results

### *n*TBP-CTPR2*n* proteins: design of mono- and multi-valent tankyrase inhibitors

We functionalised the CTPR scaffold by grafting an 8-residue hTNKS-binding consensus peptide (TBP), REAGDGEE, identified from a mutational analysis of hTNKS substrates,^36^ onto the loop between two adjacent repeats (Fig. 1A). This minimal hTNKS-binding unit was then tandemly repeated to generate a series of mono- and multi-valent molecules named *n*TBP-CTPR2*n*, with *n* between one and four (Fig. 1C). The CTPR proteins adopt a superhelical conformation with eight repeats required to complete a superhelical turn.^62^ Thus, in the *n*TBP-CTPR2*n* construct design, the TBP loops will be offset from each other by approximately 90° (Fig. 1D). All *n*TBP-CTPR2*n* constructs could be expressed and purified in high yields in *E. coli* (∼20 mg per litre of culture). All proteins were soluble in solution.

### Effect of TBP insertions on CTPR folding and stability

We first investigated the effects on protein stability of grafting the TBP sequence onto the inter-repeat loop using far-UV circular dichroism (CD). Given the high α-helical content of the *n*TBP-CTPR2*n* constructs, all CD spectra have the expected double minima at 208 nm and 222 nm similar to those observed previously for the CTPR proteins (Fig. S1A,† left),^32^ confirming that all *n*TBP-CTPR2*n* constructs were correctly folded. There was a linear increase in the molar ellipticity at 222 nm with increasing size of the CTPR construct, which further confirms that the proteins were natively folded (Fig. S1A,† right).

Next, thermal unfolding was performed to investigate the stability of the *n*TBP-CTPR2*n* constructs. We used the ellipticity at 222 nm as a measure of α-helical content. All proteins were found to be extremely thermostable, with only the smallest protein, 1TBP-CTPR2, undergoing complete denaturation. The larger *n*TBP-CTPR2*n* proteins remained partly folded even at the highest temperature, confirming that the stability increases with increasing number of repeats. The midpoint of the unfolding transition (T_M_) is therefore provided only for 1TBP-CTPR2 and corresponds to 75 °C (Fig. S1B†). Following thermal denaturation, the samples were allowed to return to 20 °C, and the CD spectra re-measured to evaluate the reversibility of the reaction. For all of the proteins, there was no significant difference between the CD spectrum before and after thermal denaturation, indicating that unfolding is reversible (Fig. S1C†).

Chemical-induced denaturation experiments were also performed, monitoring the fluorescence of the tryptophan residues (one per repeat) (Fig. S2†). A single transition was observed for all of the constructs, and the data were fitted to a two-state model to give the midpoints of unfolding (*D*_50%_), *m*-values (*m*_D–N_, a parameter related to the change in solvent-exposure upon unfolding) and the free energies of unfolding 
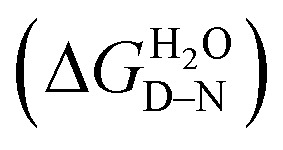
 (Table S2†). There was a significant increase in *D*_50%_ and *m*-value between 1TBP-CTPR2 and 2TBP-CTPR4 and smaller increases for the larger proteins. Lastly, the *n*TBP-CTPR2*n* showed a decrease in the stability compared with their respective counterparts without the TBP loops. As previously shown,^32^ loop insertion has a destabilizing effect through both the entropic cost of loop closure and a consequent weakened coupling of adjacent repeats.

**Fig. 2:**
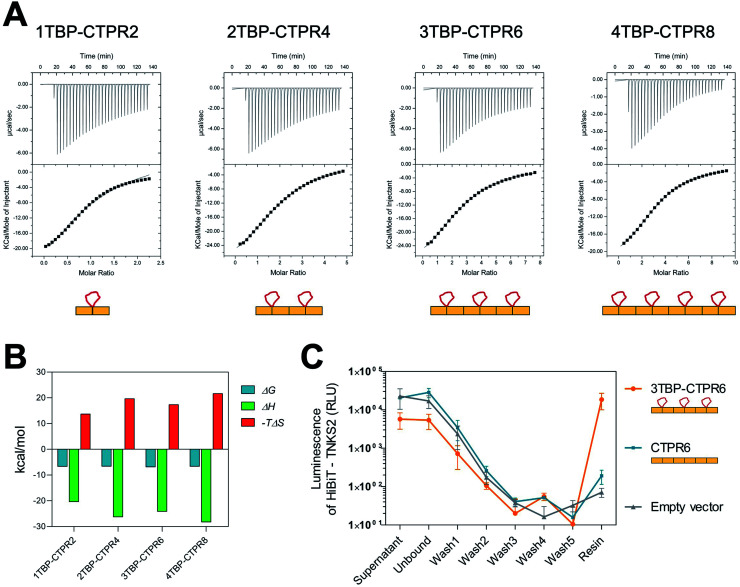
Binding of TBP-CTPR proteins to hTNKS2 *in vitro* and in the cell. (A) Representative ITC traces from left to right: hTNKS2 ARC4 (500 μM) into 1TBP-CTPR2 (50 μM), hTNKS2 ARC4 (500 μM) into 2TBP-CTPR4 (25 μM), hTNKS2 ARC4 (500 μM) into 3TBP-CTPR6 (16.6 μM), hTNKS2 ARC4 (500 μM) into 4TBP-CTPR8 (12.5 μM); experiments were performed at 25 °C. The concentrations of *n*TBP-CTPR2*n* used were chosen so that the molar ratio between the number of TBP loops and TNKS2 ARC4 protein was constant. (B) Thermodynamic parameters (Δ*G*, Δ*H* and –*T*Δ*S*) obtained by ITC for the binding of the four *n*TBP-CTPR2*n* proteins to hTNKS2 ARC4. For each parameter, the average from two independent experiments is plotted. (C) HiBiT-tagged hTNKS2 protein levels measured by luminescence intensity throughout each step of the HiBiT-qIP method. IP was performed in the presence of HA-tagged 3TBP-CTPR6, CTPR6 and an empty vector. Average values and standard deviation from two independent biological replicates are shown.

We next investigated whether all of the TBP loops in the multi-valent constructs are accessible for binding by measuring the affinity and stoichiometry for each *n*TBP-CTPR2*n* construct binding to hTNKS2 ARC4 (the fourth ankyrin-repeat cluster of hTNKS2), which contains a single binding site for the TBP (Fig. 2A). All *n*TBP-CTPR2*n* constructs showed similar low-micromolar affinities, and the stoichiometry was found to increase from 1TBP-CTPR2 to 3TBP-CTPR6 in proportion to the number of TBP loops, confirming that all sites are available for binding (Table 1). For 4TBP-CTPR8, the stoichiometry is lower than 4, likely because steric hindrance precludes binding of four hTNKS2 ARC4 molecules to one 4TBP-CTPR8 molecule. No binding could be detected for a control CTPR6 protein containing no TBP (Fig. S3†). The changes in the free energy (Δ*G*), enthalpy (Δ*H*) and entropy (–*T*Δ*S*) for the binding of the four *n*TBP-CTPR2*n* proteins to hTNKS2 ARC4 are plotted in Fig. 2B and listed in Table S3.† The results show that in all cases the interaction is enthalpy-driven. There is a slight tendency for both the enthalphic gain and entropic cost of binding to increase with increasing number of hTNKS2-binding loops. The increase in enthalpy with increasing number of binding loops is to be expected, as having multiple binding sites in close proximity should make it more likely for the protein to bind a target again after dissociation. The greater entropic cost of ordering a greater number of loops upon binding is also as expected. The greater entropic cost is offset by the greater enthalpic gain, and hence the values of Δ*G* are similar for all four *n*TBP-CTPR2*n* proteins.

**Table 1:** Stoichiometry (*N*) and dissociation constant (*K*_d_) obtained by ITC for the binding of *n*TBP-CTPR2*n* proteins to hTNKS2 ARC4. For each protein, the values listed are from two independent experiments

Protein	Stoichiometry (*N*)	*K*_d_ (μM)
1TBP-CTPR2	1.01 ± 0.03	14.68 ± 0.99
	0.90 ± 0.02	12.80 ± 1.10
2TBP-CTPR4	2.18 ± 0.02	11.89 ± 0.26
	2.05 ± 0.01	18.59 ± 0.39
3TBP-CTPR6	2.72 ± 0.03	5.62 ± 0.21
	2.77 ± 0.02	20.12 ± 0.39
4TBP-CTPR8	3.38 ± 0.01	13.61 ± 0.19
	2.91 ± 0.03	15.48 ± 0.43

### *n*TBP-CTPR2*n* proteins and hTNKS interact in the cell

Binding between hTNKS and *n*TBP-CTPR2*n* in the cell was confirmed using a modified version of the HiBiT-based quantitative immunoprecipitation (HiBiT-qIP) method, that provides a much faster and more sensitive read-out than conventional IP by western blot.^66^ The method relies on the split luciferase complementation of the two NanoLuc fragments, HiBiT and LgBiT, providing a sensitive and quantitative method to track any HiBiT-tagged protein.^67^ HA-tagged *n*TBP-CTPR2*n* was used as bait to pull-down HiBiT-tagged hTNKS2 using an anti-HA resin. The presence of HiBiT-tagged hTNKS2 was followed and quantified through the luminescence signal generated by the HiBiT technology, allowing a real-time measurement of the pulled-down target throughout the process. The results show that HiBiT-tagged hTNKS2 is pulled down by 3TBP-CTPR6 but not by the control CTPR6 construct, confirming that the grafted TBP loop mediates the binding to hTNKS2 within the cellular environment also. Moreover, no non-specific binding of HiBiT-tagged hTNKS2 to the Anti-HA Agarose resin was detected (Fig. 2C). Nano-Glo HiBiT blotting further confirmed the presence of HiBiT-tagged hTNKS2 in the eluted samples (Fig. S4†). Likewise, the bait constructs and tubulin were detected by standard western blot methods (Fig. S4†).

### Design of trimeric *n*TBP-CTPR2*n*-foldon constructs

We introduced the foldon motif at the C-terminus of each *n*TBP-CTPR2*n* construct to induce trimerisation. In this way, each homo-trimeric construct will display up to twelve TBP loops and in a different geometry compared to the corresponding monomeric constructs. The foldon is a short (30 residue) but highly stable motif of the protein fibritin from the T4 bacteriophage.^57^ The so-called ‘NCCF’ construct of fibritin contains an N-terminal trimeric coiled-coil region followed by the C-terminal foldon.^68^ Here, we introduced the foldon and part of its preceding coiled-coil domain into the *n*TBP-CTPR2*n* constructs by grafting the helical coiled-coil onto a newly introduced C-terminal CTPR helix (Fig. S5,† top). This helix corresponds to an extra helix A (*i.e.* half of one CTPR). As all α-helices share the same heptad conformation, it is possible to graft residues of a particular heptad position from a ‘guest’ helix onto a ‘host’ helix. When grafting a new site onto a CTPR helix, the native residues at the interface between the helix and the neighbouring repeat need to be preserved in order for the grafted helix to remain folded onto the rest of the CTPR scaffold. These residues were identified as Ala3, Leu7 and Ala10 of helix A, which are involved in the inter-repeat interface with the helix B of an adjacent repeat. Part of the remaining residues of helix A were substituted with the residues of the fibritin NCCF coiled-coil domain (residues 65 to 77) involved in the trimeric interface. This was followed by an insertion of the remaining C-terminal end of the coiled-coil domain and the foldon (NCCF residues 78 to 109) (Fig. S5,† top). The resulting constructs are referred as *n*TBP-CTPR2*n*-foldon, and the protein sequences are given in Table S1.† A model of the trimeric CTPR2-foldon protein is shown in Fig. 1E. This protein is expected to display the TBP loops at an angle of 120° to one another. The *n*TBP-CTPR2*n*-foldon proteins were expressed and purified as for their monomeric counterparts. Analysis by native gel electrophoresis is consistent with trimerisation of those proteins that contain the foldon domain (Fig. S5,† bottom). The series lacking the foldon domain (1TBP-CTPR2, 2TBP-CTPR4, 4TBP-CTPR8, calculated molecular weights 11.3 kDa, 20.6 kDa and 39.4 kDa, respectively) run at increasing molecular weights between the 20 kDa and 66 kDa molecular weight markers. In contrast, those with the foldon domain (1TBP-CTPR2-foldon, 2TBP-CTPR4-foldon, 4TBP-CTPR8-foldon, calculated molecular weights as trimers 49.2 kDa, 77.4 kDa and 133.5 kDa, respectively) run at higher molecular weights between the 66 kDa and 146 kDa markers.

The oligomerization was further analysed by size-exclusion chromatography coupled with multi-angle light scattering (SEC-MALS). The results show that 3TBP-CTPR6 and 3TBP-CTPR6-foldon (representative of the two types of *n*TBP-CTPR2*n* constructs) are, respectively, monomeric and trimeric in solution (Fig. S6†). The average molecular weight obtained for 3TBP-CTPR6 (30 kDa calculated molecular weight) is 38 kDa, consistent with it being monomeric. The majority of the 3TBP-CTPR6-foldon sample (105 kDa calculated molecular weight as a trimer) elutes at an average molecular weight of 131 kDa, consistent with it forming a trimer with some less-populated oligomeric species at lower and higher molecular weight also present (99, 204 and 321 kDa, respectively).

### Multivalent interactions induce the formation of large macromolecular assemblies with variable internal dynamics

**Fig. 3:**
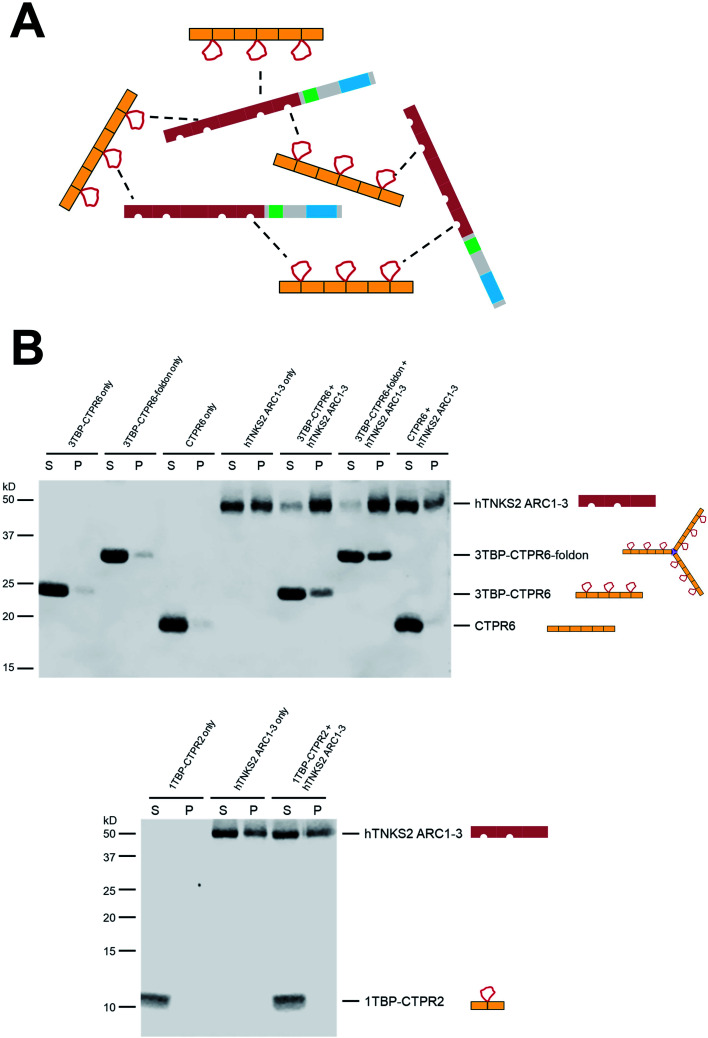
Multivalent interactions between TBP-CTPR proteins and hTNKS2 ARC1–3 analysed using a co-precipitation assay. (A) Schematic representation of the macromolecular assemblies formed by the interaction of the multivalent hTNKS2 protein and the 3TBP-CTPR6 protein (used as representatives of the multivalent CTPR arrays). (B) After centrifugation, supernatant (S) and pellet (P) were separated and run on a 12% (top) or 16% (bottom) SDS polyacrylamide gel. For co-sedimentation, proteins were mixed in equal volumes at the following concentration: 10 μM 3TBP-CTPR6 (30 μM TBP loop concentration), 3.3 μM 3TBP-CTPR6-foldon (30 μM TBP loop concentration), 10 μM CTPR6 and 10 μM hTNKS2 ARC1–3 (20 μM TBP-binding sites). Gel images were obtained using Li-COR Odyssey Fc Imaging System.

When two multivalent proteins interact, they have the potential to engage with multiple partners simultaneously, leading to the formation of large supramolecular assemblies (see for example work by Rosen and colleagues^69^). This is indeed what we observe with hTNKS2 and the multivalent tankyrase-binding CTPR proteins (schematically represented in Fig. 3A). These species could be detected using a co-precipitation assay, in which the multivalent 3TBP-CTPR6 and 3TBP-CTPR6-foldon constructs, chosen as representatives of the linear and trimeric arrays, respectively, co-precipitate in the presence of the multivalent hTNKS2 construct corresponding to the first three ARC subdomains (hTNKS2 ARC1–3). It is worth noting that, despite 3TBP-CTPR6 and 3TBP-CTPR6-foldon being incubated at the same TBP molar concentration, the trimeric 3TBP-CTPR6-foldon construct precipitated to a higher extent and lead to an increased precipitation rate of hTNKS2 ARC1–3. In contrast, the mono-valent 1TBP-CTPR2 construct and the control CTPR6 construct remained in the supernatant, confirming that multivalency of TBP loops is required for the formation of a large supramolecular assembly with multivalent hTNKS2 (Fig. 3B).

Titration of increasing concentrations of 3TBP-CTPR6 or 3TBP-CTPR6-foldon proteins into hTNKS2 ARC1–3 showed that the extent of co-precipitation was dependent on the molar ratio of the two interacting molecules, until saturation is reached. Analysis of the gel band intensities allowed us to calculate the molar concentration of each species in the pellet, providing an estimation of the stoichiometries of the macromolecular complexes that had precipitated (Fig. S7 and Table S4†). The results indicate a 1 : 1 stoichiometry for the 3TBP-CTPR6 : hTNKS2 ARC1–3 complex, and a higher stoichiometry of 1 : 2 for the 3TBP-CTPR6-foldon : hTNKS2 ARC1–3 complex, explaining why the trimeric 3TBP-CTPR6-foldon can induce a greater extent of hTNKS2 ARC1–3 co-precipitation than the linear 3TBP-CTPR6.

Next, these protein assemblies were examined using negative stain Transmission Electron Microscopy (TEM). The results shown in Fig. S8† confirm that the large, macromolecular clusters are formed only when both interacting partners are multivalent.

**Fig. 4:**
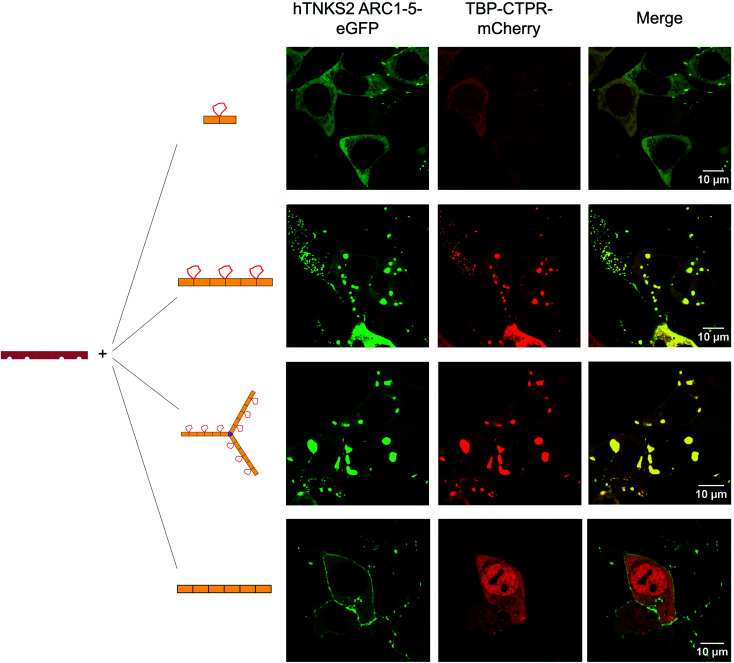
Fluorescence microscopy of HEK293T cells co-transfected with mCherry-tagged CTPR proteins and eGFP-tagged hTNKS2 ARC1–5. Co-localisation in large macromolecular clusters is observed for eGFP-tagged hTNKS2 ARC1–5 in combination with mCherry-tagged 3TBP-CTPR6 or with mCherry-tagged 3TBP-CTPR6-foldon. These clusters are not observed for the mono-valent mCherry-tagged 1TBP-CTPR2 protein or the control mCherry-tagged CTPR6 protein. Scale bars for all images are 10 μm.

**Fig. 5:**
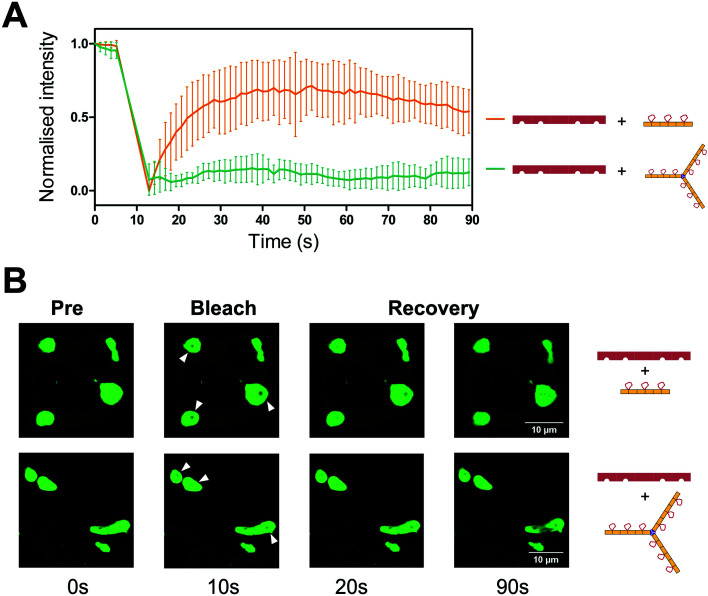
FRAP analysis of the assemblies formed between hTNKS2 ARC1–5 and TBP-CTPR proteins. (A) FRAP analysis was performed on seven individual Regions of Interest (ROI) selected within macromolecular assemblies induced by eGFP-tagged hTNKS2 ARC1–5 in complex with 3TBP-CTPR6 (orange) or 3TBP-CTPR6-foldon (green). HEK293T cells were bleached in the ROI and fluorescence recovery of eGFP-tagged hTNKS2 ARC1–5 was monitored over 90 seconds. (B) Representative ROI selected within three independent macromolecular assemblies are shown before, during and post bleaching.

To visualise the effect of multivalency inside the cell, HEK293T cells were co-transfected with an hTNKS2 construct encoding the ARC1–5 subdomains fused to eGFP (referred to as hTNKS2 ARC1–5–eGFP) and the multivalent 3TBP-CTPR6 or 3TBP-CTPR6-foldon constructs fused to mCherry. Large, polymeric species were visible in the cytoplasm, where the signals of eGFP and mCherry perfectly co-localised (Fig. 4). In agreement with the findings of the co-precipitation assays, these large assemblies were formed only when the multivalent 3TBP-CTPR6 and 3TBP-CTPR6-foldon proteins were co-expressed with hTNKS2 ARC1–5, which is also multivalent. In contrast, when the same CTPR proteins were co-expressed with a monovalent hTNKS2 construct comprising only the first ARC domain (hTNKS2 ARC1–eGFP), no polymeric assemblies were observed (Fig. S9†). Likewise, when the multivalent hTNKS2 was co-expressed with the monovalent 1TBP-CTRP2, no polymeric species were observed (Fig. 4). To further compare the properties of the macromolecular assemblies generated in the cell by 3TBP-CTPR6 and 3TBP-CTPR6-foldon bound to hTNKS2 ARC1–5, Fluorescence Recovery After Photobleaching (FRAP) was used to assess the dynamics by measuring the recovery rate of the eGFP signal (Fig. 5). The assemblies induced by 3TBP-CTPR6 showed fluorescence recovery within 30 seconds, suggesting that the proteins within the assembly are dynamic in nature. In contrast, the assemblies generated by the 3TBP-CTPR6-foldon showed no fluorescence recovery within the same timeframe, suggesting that the presence of nine hTNKS binding sites on 3TBP-CTPR6-foldon leads to the formation of a more interconnected, rigid assembly.

### *n*TBP-CTPR2*n* and *n*TBP-CTPR2*n*-foldon proteins inhibit Wnt signalling

**Fig. 6:**
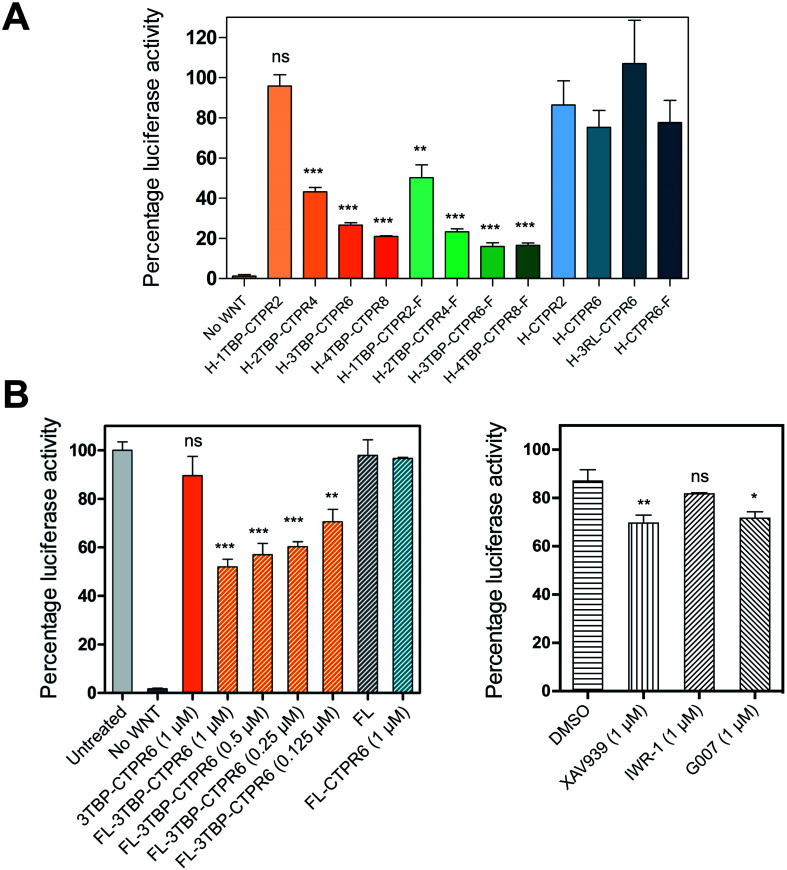
The effect on Wnt-activated HEK293T cells following treatment with the indicated HiBiT-tagged *n*TBP-CTPR2*n* constructs. (A) TOPFLASH reporter assay for HEK293T cells transfected with TCF-Firefly and Renilla reporter gene vectors and an expression vector encoding the constructs listed. For each sample, Luciferase activities were normalised with the Renilla values and the ratio was expressed as relative luciferase activity to the control well transfected with the empty HiBiT vector set at 100% (not shown in the graph). The monomeric constructs, trimeric constructs and controls are shown in different shades of orange, green and blue, respectively. F indicates the foldon motif, H indicates the N-terminal HiBiT tag. Standard deviation was calculated from triplicate sample measurements. The significance of the difference between samples (ns: non-significant, *p* > 0.05, *p ≤ 0.05 **p ≤ 0.01, ***p ≤ 0.001) was assessed using One-way ANOVA coupled with Dunnett's Multiple Correction test. 1TBP-CTPR2 and 1TBP-CTPR2-F were compared to CTPR2. Multivalent linear and trimeric constructs were compared to 1TBP-CTPR2 and 1TBP-CTPR2-F, respectively. (B) (left) The figure on the left shows the effect on Wnt signaling of treatment with fusogenic liposome-encapsulated 3TBP-CTPR6. Each treatment was with 20 μL of liposomes. In brackets are the concentrations of the proteins used. For each run, data were normalised by the untreated control well, set at 100%. Bars with diagonal stripes correspond to samples treated with liposomes. No Wnt: cells without Wnt pathway activation and not treated with liposomes. Untreated cell: cells not treated with liposomes. FL: empty liposomes. Error bars were obtained from triplicate sample measurements from two independent experiments. The same statistical analysis was performed as in (A), comparing the samples to FL-CTPR6. (B) (right) For comparison, the figure on the right shows the effect on Wnt signaling of hTNKS small molecule inhibitors used interventionally at the indicated concentration. Data were normalised relative to the untreated control well, which was set at 100% (not shown in the graph). Error bars were determined from two independent sample measurements. The same statistical analysis was performed as in (A), comparing the small molecules to DMSO.

We next evaluated the inhibitory capabilities of the monomeric and trimeric *n*TBP-CTPR2*n* constructs on Wnt signalling using the TOPFLASH reporter assay. Treatment of HEK293T cells with all the *n*TBP-CTPR2*n* constructs, with the exception of 1TBP-CTPR2, led to a reduction in Wnt pathway activity compared with the controls (empty vector, CTPR2 and CTPR6 and 3RL-CTPR6 containing a non-binding peptide named ‘RL’, random loop) (Fig. 6A). To assess the levels of *n*TBP-CTPR2*n* proteins in the cell during the TOPFLASH assay, we used N-terminally HiBiT-tagged *n*TBP-CTPR2*n* proteins. The 11-amino-acid HiBiT tag allows a more sensitive and quantitative measurement than western blot *via* the antibody-free split NanoLuc® luciferase technology (Promega). The results show that the extent of Wnt inhibition is proportional to the *n*TBP-CTPR2*n* protein levels as quantified 24 h after transfection (Fig. S10†). In particular, it is clear that the CTPR2 protein, whether in the monomeric or the trimeric format, is present at much lower levels than the larger proteins. To test whether the CTPR2 constructs are subject to proteasome-induced degradation, transfected wells were incubated with the proteasome inhibitor MG132 for 5 hours. The results show that there is an approximate 3-fold increase in protein levels in the presence of MG132, the exceptions being the two smallest constructs 1TBP-CTPR2 and CTPR2, whose levels are affected to a greater extent (Fig. S11†). Thus, the smallest, least thermodynamically stable CTPR protein is the most susceptible to proteasome-mediated degradation, and the foldon domain enhances its intracellular stability. In addition, the formation of the large macromolecular assemblies is likely to protect the multivalent hTNKS-binding CTPR proteins from proteolysis, further explaining why the monovalent 1TBP-CTPR2 and the non-binding control CTPRs do not accumulate in the cell. Lastly, we confirmed that none of the constructs showed cytotoxicity (Fig. S12†).

### Intracellular delivery of 3TBP-CTPR6 by encapsulation with fusogenic liposomes induces high levels of Wnt inhibition

Intracellular delivery of proteins is challenging.^70^ We used an encapsulation approach in which purified protein (3TBP-CTPR6) was loaded into fusogenic liposomes. Fusogenic liposomes have been shown to merge with the cell membrane and deliver their cargo directly into the cell cytoplasm.^71^ Encapsulation of 3TBP-CTPR6 in liposomes could minimise their premature degradation and adverse immune response, if any.^72^ Liposomal formulation of 3TBP-CTPR6 (FL-3TBP-CTPR6) and control liposomes without protein (FL) were prepared as described in the experimental section. Both FL and FL-3TBP-CTPR6 had highly positive surface with zeta potential (ZP) values of +126 mV and +75.7 mV, respectively, at pH 7.4 (Fig. S13†). The lower ZP of FL-3TBP-CTPR6 is due to encapsulation/association of the negatively charged 3TBP-CTPR6 (pI ∼ 4.8) with liposomes. The hydrodynamic sizes of FL and FL-3TBP-CTPR6 were similar, at ∼106 nm (Fig. S13†). FL and FL-3TBP-CTPR6 did not show any significant cytotoxicity even for amounts three times higher than those used in our experiments (Fig. S14†). To visualize intracellular delivery under the confocal microscope, 3TBP-CTPR6 was fluorescently labelled with rhodamine. Liposomes were prepared using the labelled protein and designated as FL-3TBP-CTPR6-RITC. Confocal images of HEK293T cells treated with FL-3TBP-CTPR6-RITC (Fig. S15†) clearly suggest that protein is inside the cells and is distributed throughout the cytoplasm. Cells treated with FL did not show any signal for 3TBP-CTPR6-RITC (Fig. S16†).

We next tested the effect of liposome-delivered 3TBP-CTPR6 on Wnt signaling using the TOPFLASH assay. A decrease in the TOPFLASH activity was observed, reaching around 50% after 6 hours incubation. Importantly, decreasing TOPFLASH activity correlated with increasing concentration of 3TBP-CTPR6 (Fig. 6B, left), indicating a dose-dependent inhibition of hTNKS. Treatment with free 3TBP-CTPR6 did not affect the luciferase expression levels as the protein cannot enter cells on its own. Also, FL did not alter luciferase expression, indicating that membrane fusion of liposomes does not interfere with the intracellular Wnt signalling. Further, control protein, CTPR6, lacking hTNKS-binding units and delivered using liposomes, did not change the intracellular luciferase levels.

For comparison, we also performed the TOPFLASH assay in the presence of three well-characterized small molecule hTNKS inhibitors (XAV939, IWR-1 and G007) that bind the catalytic domain of hTNKS. Critically, whereas the small molecule inhibitors are effective when used prophylactically (*i.e.* before Wnt pathway activation) (Fig. S17†), they are ineffective when used interventionally, *i.e.* after Wnt pathway activation (as would be the case in the clinic, since patients will have elevated Wnt signaling) (Fig. 6B, right). Strikingly, FL-3TBP-CTPR6 at the highest dose had a significantly greater inhibitory effect than any of the small molecule hTNKS inhibitors (Fig. 6B). This result shows that targeting the non-catalytic activity of hTNKS could be an effective treatment where small-molecule inhibitors have so far failed.

## Discussion

In this study, we created potent hTNKS inhibitors by combining target specificity with multivalency, two features that have not been explored in previous drug development efforts against this target. We demonstrate that modular CTPR proteins can be engineered to display both single as well as multiple copies of short hTNKS binding motifs by grafting the TBP onto the loops between adjacent repeats. Helical grafting was also applied to engineer trimeric CTPR constructs with enhanced multivalent capabilities. We show that CTPR proteins with one or more grafted hTNKS-binding motifs are stable, correctly folded and exceptionally active in inhibiting Wnt signalling. The effect of multivalency in both partners of the interaction – hTNKS and the designed *n*TBP-CTPR2*n* proteins – manifests in the formation of large, intracellular macromolecular assemblies with different dynamics dependent on the configuration (*i.e.* monomeric *versus* trimeric). Thus, hTNKS inhibition by these multivalent molecules is enhanced by clustering of the protein within these structures. Such a mechanism of action warrants further investigation and should be explored for future therapeutic efforts against hTNKS and other multivalent targets. Our experiments using fusogenic liposome delivery of 3TBP-CTPR6, one of our most potent molecules, further demonstrated rapid inhibition of Wnt pathway activity within 6 hours of treatment of HEK293T cells pre-stimulated with Wnt3A ligand. This is a noteworthy result that demonstrates interventional inhibition of the pathway, the scenario required for targeting tumours dependent on deregulated Wnt pathway activity *in vivo*. Moreover, the luciferase protein reporter of Wnt pathway activity has a relatively long half-life of approximately 12 hours, and therefore inhibition after 6 hours treatment likely reflects a much higher attenuation of Wnt pathway activity beyond the measured 50% inhibition.

We compared our molecules with three well-characterised small molecule hTNKS inhibitors and, in agreement with previous studies, we observed around 90% inhibition of Wnt pathway activity for two of them when used prophylactically: *i.e.* when they are applied either before Wnt pathway activation by Wnt3A ligand or in combination with it. Critically, however, to be of clinical use, such drugs will need to work interventionally; interventional treatment with these small molecule hTNKS inhibitors has been shown by our group and others to be much less effective due to a cell-intrinsic feed-forward mechanism preserving Wnt pathway activity.^73^ Thus, the effectiveness of liposome-delivered 3TBP-CTPR6 for interventional inhibition of Wnt pathway activity demonstrated here further highlights the power of our approach.

The work presented here points to the tremendous potential of the repeat-protein scaffold for the rational design of multivalent – and thereby potentially multi-specific – molecules for inhibiting PPIs: (1) First, CTPR proteins are small and ultrastable without need of disulphide bonds. As a result, they can accommodate small or even large sequence insertions yet remain folded and stable.^32^,^74^ Moreover, multiple such insertions are also possible without compromising the fold; it is doubtful whether other small single-domain scaffolds based on globular structures could do the same. (2) The second key consequence of the repeat-protein stability is that they can be produced recombinantly in very high yields. (3) Lastly, the repetitive, modular nature of the scaffold is a particularly unique selling point; we have shown here that we can exploit these characteristics to display one or multiple binding motifs in a precise and programmable manner. It has been estimated that there are around 100 000 such SLiMs in the human proteome,^75^ each one of which provides a potential starting point for drug discovery. The simple cut-and-paste approach used here, requiring little or no *in silico* design procedures, could be applied to harness some of these SLiMs, and the platform thus has potential to be used as a synthetic tool in diverse applications ranging from target validation to PPI inhibition, pathway modulation and ultimately molecular therapeutics.

Our confidence in the broad applicability of the scaffold to the display of diverse SLiMs arises from the finding that we could effectively graft two SLiMs with very different binding conformations onto the CTPR loops. The Keap1-binding peptide from Nrf2 adopts a tight turn-like conformation,^33^ whereas the TBP in this work binds to ARC domains in a highly extended conformation.^36^,^55^

It might appear that a limiting factor in this multivalent platform would be the potential for steric clashes between target and scaffold as well as between targets in a multivalent display. The first challenge should be approached on a case-by-case basis, as it is common to all peptide display methodology. The second problem can be mitigated by exploiting the modular nature of this technology. For example, the ITC data show that both 3TBP-CTPR6 and 4TBP-CTPR8 bind to approximately three hTNKS2 ARC4 molecules, suggesting that a maximum has been reached. We limited our grafting to one peptide for every two repeats, but peptides could be set further apart by increasing the number of intervening repeats. The rigidity of the CTPR scaffold ultimately works as a molecular ruler of predefined length and pitch. Furthermore, we have shown that trimerisation by means of the small foldon domain enhances the multivalent capability of the CTPR scaffold, presumably by providing more degrees of freedom for the formation of macromolecular complexes. Future work will focus on building bi- and multi-functional CTPR arrays using multiple SLiMs to bring two or more different targets together in a predefined geometry, for example to redirect enzyme activity, alter sub-cellular localisation or reprogramme signaling pathways.

## Experimental section

### Molecular biology

The gene encoding 1TBP-CTPR2 was synthesised by Integrated DNA Technologies. It was designed with appropriate restriction sites at each terminus to allow ligation and the generation of multivalent constructs by concatemerisation.^76^ We used a variant of the original CTPR sequence developed by Regan and coworkers modified at two positions (D18Q and E19K)^63^,^64^ to further enhance the stability of the CTPR scaffold. Our CTPR proteins do not have the extra C-terminal helix that has been used by some groups to improve solubility (Fig. 1B).^25^,^31^,^77^,^78^ Likewise, the gene encoding the foldon motif was synthesized and ligated at the 3′ end of each *n*TBP-CTPR2*n* construct using the appropriate restriction enzymes (Thermo Fisher Scientific). The genes encoding hTNKS2 fragments were also amplified and ligated using the same method. Different combinations of tags (His, HA, HiBiT, eGFP, mCherry) were fused at the N- or C-terminal end of each construct. Genes were ligated into a modified pRSRET B vector (Thermo Fisher Scientific) for bacterial expression, or into the pcDNA3.1(–) vector (Thermo Fisher Scientific) for mammalian expression. Correct clones were confirmed by DNA sequencing (Eurofins Genomics). Protein sequences of all of the generated CTPR constructs are listed in Table S1.† An *E. coli* expression plasmid encoding TNKS2 ARC4 was previously generated.^55^

### Protein purification

The pRSET B vectors encoding the His-tagged *n*TBP-CTPR2*n* constructs were transformed into chemically competent *Escherichia coli* C41 cells. Colonies were grown at 37 °C in 2xYT media (Formedium) containing ampicillin (50 μg mL^–1^), shaking at 220 rpm until the optical density at 600 nm was ∼0.6. Protein expression was induced with 0.5 mM isopropyl β-d-1-thiogalactopyranoside (IPTG) for 16–20 hours at 20 °C. Cells were harvested and resuspended in buffer A (50 mM Tris–HCl, 150 mM NaCl, pH 8.0) including EDTA-free SIGMAFAST protease inhibitors (Sigma-Aldrich), Lysozyme and DNase I. Cells were lysed by high-pressure homogenization using an Emulsiflex C5 homogenizer (Avestin) at 15 000 psi, and cell debris was removed by centrifugation steps at 40 000*g* for 40 minutes. *n*TBP-CTPR2*n* constructs were purified by immobilised metal ion affinity chromatography (IMAC) on a 5 mL HisTrap Excel column according to the manufacturer's instructions (Cytiva). The column was washed with 20 CV of buffer A containing 20 mM imidazole to prevent nonspecific interaction of lysate proteins to the beads. Proteins were eluted with buffer B (50 mM Tris–HCl, 150 mM NaCl, 500 mM imidazole, pH 8.0). All proteins were subsequently purified by size-exclusion using a HiLoad 16/600 Superdex 75 pg column (Cytiva) pre-equilibrated in buffer A. Purified protein was flash-frozen and stored at –80 °C until further use.

Proteins to be analysed by size-exclusion chromatography coupled with multi-angle light scattering (SEC-MALS) were further purified by SEC using a Superdex 200 10/300 GL (Cytiva) preequilibrated in buffer A. Purified protein samples (100 μL) were subjected to SEC-MALS, performed on a SEC Superdex 200 increase 10/300 GL column (Cytiva) preequilibrated in buffer A, in line with a multi-angle light scattering module (DAWN-8+; Wyatt Technologies) and a differential refractometer (Optilab T-rEX; Wyatt Technologies). The light scattering and protein concentration at each point across the peaks in the chromatograph were used to determine the absolute molecular mass from the intercept of the Debye plot using Zimm's model as implemented in the ASTRA v7.3.0.11 software (Wyatt Technologies). To determine interdetector delay volumes, band-broadening constants and detector intensity normalization constants for the instrument, BSA (Thermo Fisher Scientific) was used as a standard prior-to sample measurement. Data were plotted with the program PRISM 8 (GraphPad Software Inc.).

His-tagged Tankyrase-2 Ankyrin Repeat Cluster 4 (residues 488-649, referred to as hTNKS2 ARC4) and Tankyrase-2 Ankyrin Repeat Clusters 1–3 (residues 2-485, referred to as hTNKS2 ARC1–3) were expressed and purified as reported above and previously,^55^ with all buffers containing 2 mM DTT. To remove the His tag, proteins were incubated with 125 U of thrombin (Sigma-Aldrich) overnight at RT on a rotating mixer. Cleaved protein was further run over IMAC, and the flow-through was collected. The final purity and identity of all the proteins were determined by SDS-PAGE and MALDI mass spectrometry (PNAC, Department of Biochemistry, Cambridge, UK), respectively. Concentrations were determined by absorbance at 280 nm using the calculated extinction coefficient (ExPaSy ProtParam)^79^ for each protein.

### Circular dichroism spectroscopy (CD) and thermal denaturation experiments

All protein samples were prepared in 10 mM sodium phosphate buffer, 150 mM NaCl, pH 7.4, at a final concentration of 20 μM. Circular dichroism (CD) measurements were performed on a Chirascan CD spectrophotometer (Applied Photophysics) at 20 °C. CD spectra were acquired at wavelengths between 200 nm and 280 nm using a 1 nm bandwidth at a scan speed of 120 nm per minute. Readings were repeated in triplicate and the data averaged. Thermal denaturation experiments were carried out by increasing the temperature of the protein samples from 20 °C to 92 °C in 1 °C steps, and the ellipticity at 222 nm was monitored. Readings were repeated five times, averaged and fitted to a sigmoidal Boltzmann equation including a sloping baseline term using PRISM (GraphPad Software Inc.). Subsequently, the sample was allowed to cool back to 20 °C and the CD spectrum was re-acquired.

### Equilibrium denaturation monitored by fluorescence spectroscopy

High-throughput equilibrium denaturation experiments were performed as previously described.^74^ All protein samples (at concentrations between 6–10 μM) were prepared in 50 mM sodium phosphate buffer, 150 mM NaCl, pH 6.8. Protein samples were dispensed into 96-well, half-area, black polystyrene plates (Corning) using a Microlab ML510B dispenser (Hamilton) containing different concentrations of guanidinium hydrochloride (GdmHCl) between 0 M and 6 M in increments of 0.1 M per well. Plates were covered with 96-well Microplate Aluminium Sealing Tape (Corning) to avoid evaporation and incubated at 25 °C for 1 hour. Plate measurements were carried out on a CLARIOstar Plate Reader (BMG LABTECH) with a tryptophan-detection filter set consisting of an excitation filter (275–285 nm), a dichroic PL325 nm, and an emission filter (350–370 nm) at 25 °C. Each measurement was performed in triplicate. The plots of fluorescence intensity *versus* denaturant concentration were fitted to a two-state model with sloping baselines, in which only fully folded and fully unfolded states are populated, using the following equation:^80^
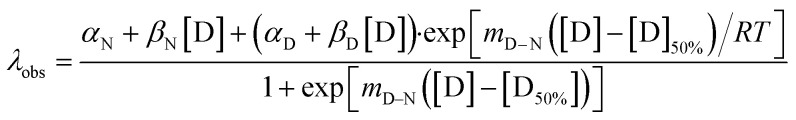
where *a*_N_ and *a*_D_ are the state signal at the lowest and highest concentration of denaturant, respectively, *β*_N_ and *β*_D_ are the slopes of the native and unfolded state baseline, respectively, *m*_D–N_ is a constant related to the change in solvent accessible surface area upon unfolding, [D] is the denaturant concentration and [D]_50%_ is the midpoint of the unfolding transition. The free energy of unfolding, 
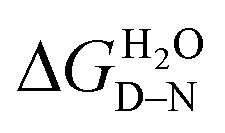
, was calculated as the product of the midpoint of unfolding ([D]_50%_) and the *m*-value (*m*_D–N_), a constant proportional to the surface area exposed upon unfolding.

### Isothermal titration calorimetry (ITC)

ITC was performed at 25 °C using a MicroCal VP-ITC (Malvern Panalytical). Proteins were dialysed into 10 mM sodium phosphate buffer, 150 mM NaCl, 0.5 mM TCEP, pH 7.4. TNKS2 ARC4 was titrated into the sample cell containing one of the *n*TBP-CTPR2*n* constructs. Injections of TNKS2 ARC4 into the cell were initiated with one injection of 5 μL over 6 seconds, followed by 29 injections of 10 μL over 12 seconds. Raw data were first subjected to baseline determination using NITPIC software^81^ and were fitted using the OneSite model within Origin 7.0 software to a non-linear regression.

### Immunoprecipitation (IP)

HEK293T cells were cultured in 15 mL cell growth media (Dulbecco's Modified Eagles Medium (DMEM), high glucose, pyruvate, (Thermo Fisher Scientific) supplemented with 10% Fetal Bovine Serum (Sigma-Aldrich) and 1× Penicillin/Streptomycin (Gibco). HEK293T cells in 10 cm dishes were transfected with 3.5 μg of pcDNA3.1(–) vector encoding HiBiT-tagged hTNKS2 and 3.5 μg of pcDNA3.1(–) vector encoding a HA-tagged 3TBP-CTPR6, CTPR6 or empty vector. Transfection was performed using Lipofectamine 2000 transfection reagent according to the manufacturer's protocol (Thermo Fisher Scientific). Following 48 hours from transfection, cells were washed twice in PBS and lysed for 30 minutes in 1 mL cold lysis buffer (50 mM Tris–HCl, 225 mM KCl, 1% NP-40, pH 7.5) including protease inhibitor cocktail (Sigma-Aldrich), 10 mM NaF, 1 mM Na_3_VO_4_, 1 mM PMSF. Cell debris was removed by centrifugation at 16 000*g* for 20 minutes. IP of HiBiT-tagged hTNKS2 bound to HA-tagged 3TBP-CTPR6 was performed by incubating the supernatant for 4 hours with 20 μL monoclonal anti-HA Agarose (Sigma-Aldrich), pre-equilibrated in lysis buffer. Anti-HA agarose resin was washed 4 times with 500 μL lysis buffer. Elution was performed by adding 20 μL 2× loading dye containing denaturant SDS to the settled resin. All steps following cell washing were performed at 4 °C. The presence of HiBiT-tagged hTNKS2 throughout the IP process was detected by mixing 5 μL of each sample with the Nano-Glo HiBiT Lytic Detection System according to the manufacturer's instructions (Promega). The amount of HiBiT-tagged hTNKS2 bound to the beads was quantified by mixing 5 μL of 50% anti-HA Agarose resin slurry with the Nano-Glo HiBiT Lytic Detection System before elution. Data were obtained from two biological replicates.

Samples for western blot were transferred from a 10% polyacrylamide gel to a nitrocellulose membrane (pore size 0.20 μm, Pharmacia Biotech). HiBiT-tagged hTNKS2 was visualised using the Nano-Glo HiBiT Blotting System according to the manufacturer's instructions (Promega). HA-tagged 3TBP-CTPR6 and CTPR6 were detected using anti-HA-Tag (C29F4, 1 : 1000 dilution) rabbit monoclonal antibody (cell signaling). Tubulin was identified using anti-alpha Tubulin antibody (ab7291, 1 : 10 000 dilution) mouse monoclonal antibody (Abcam). HRP-conjugated goat anti-mouse (P0447, 1 : 10 000 dilution) and swine anti-rabbit (P0399, 1 : 10 000 dilution) were used as secondary antibodies (Dako). The membrane was developed using Amersham ECL western blotting Detection Reagent and ECL select western blotting Detection Reagent (Cytiva) on a LI-COR Odyssey Fc Imaging System.

### Co-precipitation assay

20 μL of 10 μM hTNKS2 ARC1–3 was mixed with an equal volume of 1TBP-CTPR2, 3TBP-CTPR6, 3TBP-CTPR6-foldon or CTPR6 at the indicated concentration in co-precipitation buffer (50 mM Tris–HCl, 150 mM NaCl, 0.5 mM TCEP, pH 7.3). Samples were incubated at room temperature for 1 hour and centrifuged at 20 000*g* for 30 minutes at room temperature. The supernatant was collected and the pellet resuspended in an equal volume of buffer. Samples were run on a polyacrylamide gel, and the gels were imaged using the Li-COR Odyssey Fc Imaging System and protein band densities were analysed using the Image Studio Lite 5.2 software.

### Fluorescence microscopy and FRAP

1 × 10^5^ HEK293T cells were seeded overnight in 700 μL of cell growth media in a μ-Slide (Ibidi). The following day, cells were transfected with 400 ng of m-Cherry tagged *n*TBP-CTPR2*n*, *n*TBP-CTPR2*n*-foldon or the control CTPR construct in combination with 600 ng of eGFP-tagged TNKS2 ARC1–5 or 100 ng of TNKS2 ARC1, using 1.75 μL lipofectamine 2000 transfection reagent according to the manufacturer's protocol (Thermo Fisher Scientific). Following 48 hours from transfection, cells were imaged on a Leica TCS SP5 confocal microscope with a 100× oil-immersion objective lens (1.4 numerical aperture). Excitation and filters were as follows: eGFP, excitation at 488 nm, emission from 500–540 nm; mCherry, excitation at 543 nm, emission from 600–630 nm. For FRAP, individual circular regions of interest (ROI) were bleached using the 488 laser at 100% power for 5 seconds. Fluorescence intensity changes were recorded comparing 5 pre-bleaching frames with 60 post-bleaching frames (1.3 seconds per frame). Data were analysed using Leica LAS AF suite software and data were normalised.

### Transmission electron microscopy (TEM)

hTNKS2 ARC1–3 was mixed with an equal volume and concentration (5 μM) of 1TBP-CTPR2, 3TBP-CTPR6, 3TBP-CTPR6-foldon, CTPR6 or co-precipitation buffer. Samples were incubated at room temperature for 1 hour. They were then dispensed on carbon support film, 400 mesh, 3 mm nickel grids (EM Resolutions Ltd, Saffron Walden, UK) and stained with 2% uranyl acetate (w/v). The samples were imaged on a FEI Tecnai G2 transmission electron microscope in the Cambridge Advanced Imaging Centre (CAIC, University of Cambridge, Cambridge, UK). Images were analysed using the SIS Megaview II Image Capture system.

### TOPFLASH dual-luciferase reporter assay of activity of transfected CTPR constructs

Wnt pathway activity was induced by treating cells with conditioned media obtained from L-cells expressing Wnt3A (ATCC CRL-2647) for 8 days, according to ATCC guidelines. For the TOPFLASH assay, HEK293T cells in 24-well plates were transfected with 100 ng of TCF7L2-Firefly plasmid, 10 ng of CMV-Renilla plasmid and 100 ng of pcDNA3.1(–) vector encoding HiBiT-tagged *n*TBP-CTPR2*n*, *n*TBP-CTPR2*n*-foldon or the control CTPR constructs using 0.5 μL lipofectamine 2000 transfection reagent according to the manufacturer's protocol (Thermo Fisher Scientific). Transfected cells were allowed to recover in cell growth media for 8 hours, followed by treatment with Wnt-conditioned media (derived from L-cells expressing Wnt3A; ATCC CRL-2647) at a 1 : 1 ratio for a further 16 hours. The TOPFLASH assay was performed using the Dual-Luciferase Reporter Assay System (Promega), as previously described.^82^ Relative luciferase values were obtained from three independent experiments by dividing the Firefly luciferase values by the Renilla luciferase values. The luminescence intensity of each HiBiT-tagged construct was measured by mixing 20 μL of the same cell lysate with the Nano-Glo HiBiT Lytic Detection System according to the manufacturer's instructions (Promega).

### Measurements of cellular levels of HiBiT-tagged CTPR proteins

HEK293T cells in 96-well plates were transfected with 30 ng of pcDNA3.1(–) vector encoding HiBiT-tagged *n*TBP-CTPR2*n*, *n*TBP-CTPR2*n*-foldon or the control CTPR constructs using Lipofectamine 2000 transfection reagent according to the manufacturer's protocol (Thermo Fisher Scientific). Following 19 hours incubation, cells were treated with 10 μM MG132 (Calbiochem) for 5 hours. The number of viable cells was measured using the CellTiter-Fluor Cell Viability Assay (Promega) according to the manufacturer's instructions. HiBiT-tagged constructs were quantified using the Nano-Glo HiBiT Lytic Detection System (Promega) as above. HiBiT values obtained from three independent replicates were normalised using the corresponding cell viability measurements.

### Liposomal formulation of 3TBP-CTPR6 protein

Fusogenic Liposomes (FL) containing 3TBP-CTPR6 (FL-3TBP-CTPR6) were prepared using a previously reported method.^71^ In a typical procedure, 250 μg of 1,2-dioleoyl-3-trimethylammonium-propane (DOTAP), 250 μg of 1,2-Dioleoyl-*sn*-glycero-3-phosphoethanolamine (DOPE), and 25 μg of 1,1′-dioctadecyl-3,3,3′,3′-tetramethylindotricarbocyanine iodide (DiR) (Avanti Polar lipids) were dissolved in 250 μL of chloroform (Merck). The solution was dried overnight inside a vacuum desiccator and the resulting lipid film was hydrated with 125 μL of 25 μM 3TBP-CTPR6 (in 10 mM HEPES, pH 7.4). This dispersion was first vortexed for 2 minutes and then ultrasonicated for 20 minutes at room temperature. Control FL were prepared similarly by hydrating the lipid film with 125 μL of 10 mM HEPES, pH 7.4. The liposomes were stored at 4 °C until further use. The zeta potential and hydrodynamic size of the FL were measured at 25 °C and pH 7.4 in 10 mM HEPES buffer using a Zetasizer Nano ZS (Malvern Instruments).

### Intracellular delivery of liposome-encapsulated 3TBP-CTPR6 protein

To directly visualise intracellular protein delivery using confocal microscopy, 3TBP-CTPR6 was labelled with rhodamine B isothiocyanate (RITC) (Sigma). In a typical procedure, 50 μL of RITC (1 mg mL^–1^ in DMSO) was slowly added to a 1 mL solution of 3TBP-CTPR6 (2 mg mL^–1^) in 0.1 M sodium carbonate buffer pH 9.0, with stirring. The solution was stirred at 4 °C for 8 hours and ammonium chloride (Sigma) was added to a final concentration of 50 mM and stirring was continued for a further 2 hours. RITC labelled 3TBP-CTPR6 (3TBP-CTPR6-RITC) was isolated from unreacted RITC using a PD10 desalting column (Cytiva). Liposomal formulation of 3TBP-CTPR6-RITC (FL-3TBP-CTPR6-RITC) was performed as described above. 1.4 × 10^5^ HEK293T cells were seeded overnight in 700 μL of cell growth media in a μ-Slide (Ibidi). The following day, medium was replaced with 700 μL cell growth media without FBS and containing 28 μL of FL-3TBP-CTPR6-RITC. Samples were incubated for 15 minutes at 37 °C and then washed twice with 1× PBS. Confocal images were acquired using a Leica TCS SP5 confocal microscope.

### Cell viability assay

4 × 10^4^ HEK293T cells were seeded for 24 hours in 100 μL of cell growth media in a 96-well plate. Cells were incubated with 100 μL liposomes in cell growth media without FBS and containing different volumes (1–12 μL) of FL and FL-3TBP-CTPR6 for 15 minutes at 37 °C. After washing twice with 1× PBS, 100 μL of CellTiter-Glo Reagent (Promega) was added, and luminescence was measured using a Clariostar microplate reader (BMG LABTECH) according to the manufacture's protocol. Untreated cells were used as control. Data were obtained from triplicate samples, and the standard deviation was calculated from two independent experiments.

### TOPFLASH assay of cellular activity of liposome-encapsulated 3TBP-CTPR6 protein and small molecule hTNKS inhibitors

HEK293T cells were seeded in 24-well plates as described above. Cells were transfected with 100 ng of TCF7L2-Firefly plasmid, 10 ng of CMV-Renilla plasmid per well using Lipofectamine 2000 transfection reagent according to the manufacturer's protocol (Thermo Fisher Scientific). Transfected cells were allowed to recover in cell growth media for 8 hours, and treated with Wnt3A-conditioned media, as above. 16 hours post Wnt pathway activation, proteins were delivered into the cells by liposomal treatment. Cells were incubated with cell growth media minus FBS containing 20 μL of liposomes, for 15 minutes at 37 °C. Following one wash in 1× PBS, Wnt3A conditioned media was added and cells were incubated for 6 hours. The TOPFLASH assay was performed, as described above, in triplicate (from two independent experiments).

For the dose-dependence analysis, the volume of liposome added was kept constant (20 μL) and the protein concentration was varied. The FL-3TBP-CTPR6 samples were prepared in the following way: lipid cakes were hydrated with 10 mM HEPES pH 7.4 and 3.125 μM, 6.25 μM, 12.5 μM, and 25 μM 3TBP-CTPR6. 20 μL of these liposomes in 500 μL cell growth media resulted in 3TBP-CTPR6 concentrations of 0.125 μM, 0.25 μM, 0.5 μM and 1 μM respectively. Samples of the liposome-encapsulated unfunctionalised control protein, referred to as FL-CTPR6, were prepared by hydrating lipid cake with 25 μM CTPR6 in 10 mM HEPES pH 7.4.

For testing the small molecule hTNKS inhibitors XAV939, IWR-1 and G007 in the TOPFLASH assay, cells were treated prophylactically with inhibitors mixed with Wnt-conditioned media and incubated for 16 hours, or interventionally with inhibitors added after overnight treatment with Wnt-conditioned media and incubated for another 6 hours. All small molecule hTNKS inhibitors were used at a final concentration of 1 μM in 0.5% DMSO. The TOPFLASH assay was then completed as described above.

## Conflicts of interest

Authors APR, LSI and PJER are inventors on patent application PCT/EP2018/068580. Authors APR, LSI and PJER are founders of PolyProx Therapeutics Limited (company number 11664980).

## Supplementary Material

Supplementary information
