# Supplementary material for: Engineering mono- and multi-valent inhibitors on a modular scaffold
Source: Chem Sci. 2020 Dec 17;12(3):880–95. doi: 10.1039/d0sc03175e (PMC7885266; doi:10.1039/d0sc03175e)

Electronic Supplementary Material (ESI) for Chemical Science.
This journal is © The Royal Society of Chemistry 2020

Supporting Information

Engineering mono- and multi-valent inhibitors on a modular scaffold

Aurora Diamante, Piyush K. Chaturbedy, Pamela J. E. Rowling, Janet R. Kumita, Rohan S. Eapen, Stephen H. McLaughlin, Marc de la Roche, Albert Perez-Riba & Laura S. Itzhaki

Table S1. Amino acid sequences of the protein constructs generated in this study.

Amino acid sequences of the $_{n}$TBP-CTPR$_{2n}$ proteins, $_{n}$TBP-CTPR$_{2n}$-foldon proteins and control proteins are listed. The TBP (tankyrase-binding peptide) is in red, the RL (random loop sequence) in green and the foldon motif is in purple. Schematic representations of each construct are also shown.

	**Sequence**	
**1TBP-CTPR2**	AEAWYNLGNAYYKQGDYQKAIEYYQKALELDPNN<br>REAGDGEEDPRSAEAWYNLGNAYYKQGDYQKAIEY<br>YQKALELDPRS	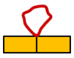
**2TBP-CTPR4**	AEAWYNLGNAYYKQGDYQKAIEYYQKALELDPNN<br>REAGDGEEDPRSAEAWYNLGNAYYKQGDYQKAIEY<br>YQKALELDPRSAEAWYNLGNAYYKQGDYQKAIEY<br>YQKALELDPNNREAGDGEEDPRSAEAWYNLGNAYY<br>KQGDYQKAIEYYQKALELDPRS	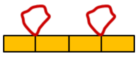
**3TBP-CTPR6**	AEAWYNLGNAYYKQGDYQKAIEYYQKALELDPNN<br>REAGDGEEDPRSAEAWYNLGNAYYKQGDYQKAIEY<br>YQKALELDPRSAEAWYNLGNAYYKQGDYQKAIEY<br>YQKALELDPNNREAGDGEEDPRSAEAWYNLGNAYY<br>KQGDYQKAIEYYQKALELDPRSAEAWYNLGNAYY<br>KQGDYQKAIEYYQKALELDPNNREAGDGEEDPRSA<br>EAWYNLGNAYYKQGDYQKAIEYYQKALELDPRS	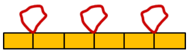
**4TBP-CTPR8**	AEAWYNLGNAYYKQGDYQKAIEYYQKALELDPNN<br>REAGDGEEDPRSAEAWYNLGNAYYKQGDYQKAIEY<br>YQKALELDPRSAEAWYNLGNAYYKQGDYQKAIEY<br>YQKALELDPNNREAGDGEEDPRSAEAWYNLGNAYY<br>KQGDYQKAIEYYQKALELDPRSAEAWYNLGNAYY<br>KQGDYQKAIEYYQKALELDPNNREAGDGEEDPRSA<br>EAWYNLGNAYYKQGDYQKAIEYYQKALELDPRSAE<br>AWYNLGNAYYKQGDYQKAIEYYQKALELDPNNRE<br>AGDGEEDPRSAEAWYNLGNAYYKQGDYQKAIEYY<br>QKALELDPRS	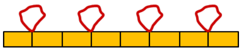
**1TBP-CTPR2-foldon**	AEAWYNLGNAYYKQGDYQKAIEYYQKALELDPNN<br>REAGDGEEDPRSAEAWYNLGNAYYKQGDYQKAIEY<br>YQKALELDPRSAKASLNLANADIKTIQEAGYIPEAPR<br>DGQAYVRKDGEWVLLSTFLRS	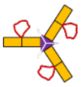
**2TBP-CTPR4-foldon**	AEAWYNLGNAYYKQGDYQKAIEYYQKALELDPNN<br>REAGDGEEDPRSAEAWYNLGNAYYKQGDYQKAIEY<br>YQKALELDPRSAEAWYNLGNAYYKQGDYQKAIEY<br>YQKALELDPNNREAGDGEEDPRSAEAWYNLGNAYY<br>KQGDYQKAIEYYQKALELDPRSAKASLNLANADIKT<br>IQEAGYIPEAPRDGQAYVRKDGEWVLLSTFLRS	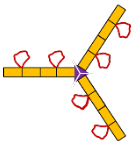

3TBP-CTPR6-foldon

AEAWYNLGNAYYKQGDYQKAIEYYQKALELDPNN
REAGDGEEDPRSAEAWYNLGNAYYKQGDYQKAIEY
YQKALELDPRSAEAWYNLGNAYYKQGDYQKAIEY
YQKALELDPNNREAGDGEEDPRSAEAWYNLGNAYY
KQGDYQKAIEYYQKALELDPRSAEAWYNLGNAYY
KQGDYQKAIEYYQKALELDPNNREAGDGEEDPRSA
EAWYNLGNAYYKQGDYQKAIEYYQKALELDPRSA
KASLNLANADIKTIQEAGYIPEAPRDGQAYVRKDGE
WVLLSTFLRS

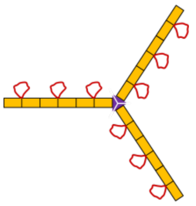

4TBP-CTPR8-foldon

AEAWYNLGNAYYKQGDYQKAIEYYQKALELDPNN
REAGDGEEDPRSAEAWYNLGNAYYKQGDYQKAIEY
YQKALELDPRSAEAWYNLGNAYYKQGDYQKAIEY
YQKALELDPNNREAGDGEEDPRSAEAWYNLGNAYY
KQGDYQKAIEYYQKALELDPRSAEAWYNLGNAYY
KQGDYQKAIEYYQKALELDPNNREAGDGEEDPRSA
EAWYNLGNAYYKQGDYQKAIEYYQKALELDPRSAE
AWYNLGNAYYKQGDYQKAIEYYQKALELDPNNRE
AGDGEEDPRSAEAWYNLGNAYYKQGDYQKAIEYY
QKALELDPRSAKASLNLANADIKTIQEAGYIPEAPRD
GQAYVRKDGEWVLLSTFLRS

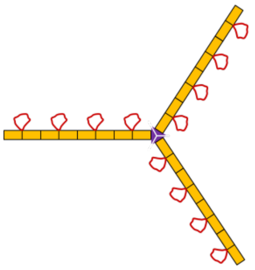

CTPR2

AEAWYNLGNAYYKQGDYQKAIEYYQKALELDPRS
AEAWYNLGNAYYKQGDYQKAIEYYQKALELDPRS

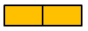

CTPR3

AEAWYNLGNAYYKQGDYQKAIEYYQKALELDPRS
AEAWYNLGNAYYKQGDYQKAIEYYQKALELDPRS
AEAWYNLGNAYYKQGDYQKAIEYYQKALELDPRS

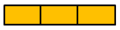

CTPR4

AEAWYNLGNAYYKQGDYQKAIEYYQKALELDPRS
AEAWYNLGNAYYKQGDYQKAIEYYQKALELDPRS
AEAWYNLGNAYYKQGDYQKAIEYYQKALELDPRS
AEAWYNLGNAYYKQGDYQKAIEYYQKALELDPRS

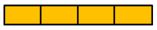

CTPR6

AEAWYNLGNAYYKQGDYQKAIEYYQKALELDPRS
AEAWYNLGNAYYKQGDYQKAIEYYQKALELDPRS
AEAWYNLGNAYYKQGDYQKAIEYYQKALELDPRS
AEAWYNLGNAYYKQGDYQKAIEYYQKALELDPRS
AEAWYNLGNAYYKQGDYQKAIEYYQKALELDPRS
AEAWYNLGNAYYKQGDYQKAIEYYQKALELDPRS

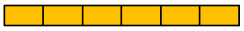

3RL-CTPR6

AEAWYNLGNAYYKQGDYQKAIEYYQKALELDPNN
GSDDPRGSRSAEAWYNLGNAYYKQGDYQKAIEYYQ
KALELDPRSAEAWYNLGNAYYKQGDYQKAIEYYQ
KALELDPNNGSDDPRGSRSAEAWYNLGNAYYKQGD
YQKAIEYYQKALELDPRSAEAWYNLGNAYYKQGD
YQKAIEYYQKALELDPNNGSDDPRGSRSAEAWYNL
GNAYYKQGDYQKAIEYYQKALELDPRS

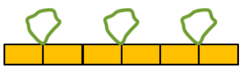

CTPR6-foldon

AEAWYNLGNAYYKQGDYQKAIEYYQKALELDPRS
AEAWYNLGNAYYKQGDYQKAIEYYQKALELDPRS
AEAWYNLGNAYYKQGDYQKAIEYYQKALELDPRS
AEAWYNLGNAYYKQGDYQKAIEYYQKALELDPRS
AEAWYNLGNAYYKQGDYQKAIEYYQKALELDPRS
AEAWYNLGNAYYKQGDYQKAIEYYQKALELDPRS
AKASLNLANADIKTIQEAGYIPEAPRDGQAYVRKDG
EWVLLSTFLRS

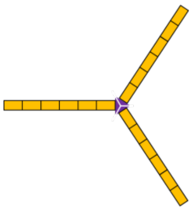

Figure S1. CD spectroscopy of $_{n}$TBP-CTPR$_{2n}$ proteins.

(A) Left: Far-UV CD spectra of 1TBP-CTPR2, 2TBP-CTPR4, 3TBP-CTPR6 and 4TBP-CTPR8. Protein concentration was 20 µM in 10 mM sodium phosphate pH 7.4, 150 mM NaCl at 20 °C. **Right:** Plot of molar ellipticity at 222 nm, obtained from the experiments shown in Fig. 2A, against the number of CTPRs in the protein (monomeric proteins only: 1TBP-CTPR2, 2TBP-CTPR4, 3TBP-CTPR6 and 4TBP-CTPR8).

(B) Thermal denaturation curves of the samples in (**A**) monitored by CD.

© CD spectra of the proteins before and after thermal denaturation (TD). Far-UV CD spectra of 1TBP-CTPR2, 2TBP-CTPR4, 3TBP-CTPR6 and 4TBP-CTPR8. All proteins were at 20 µM concentration in 10 mM sodium phosphate pH 7.4, 150 mM NaCl, and measurements were made at 20 °C.

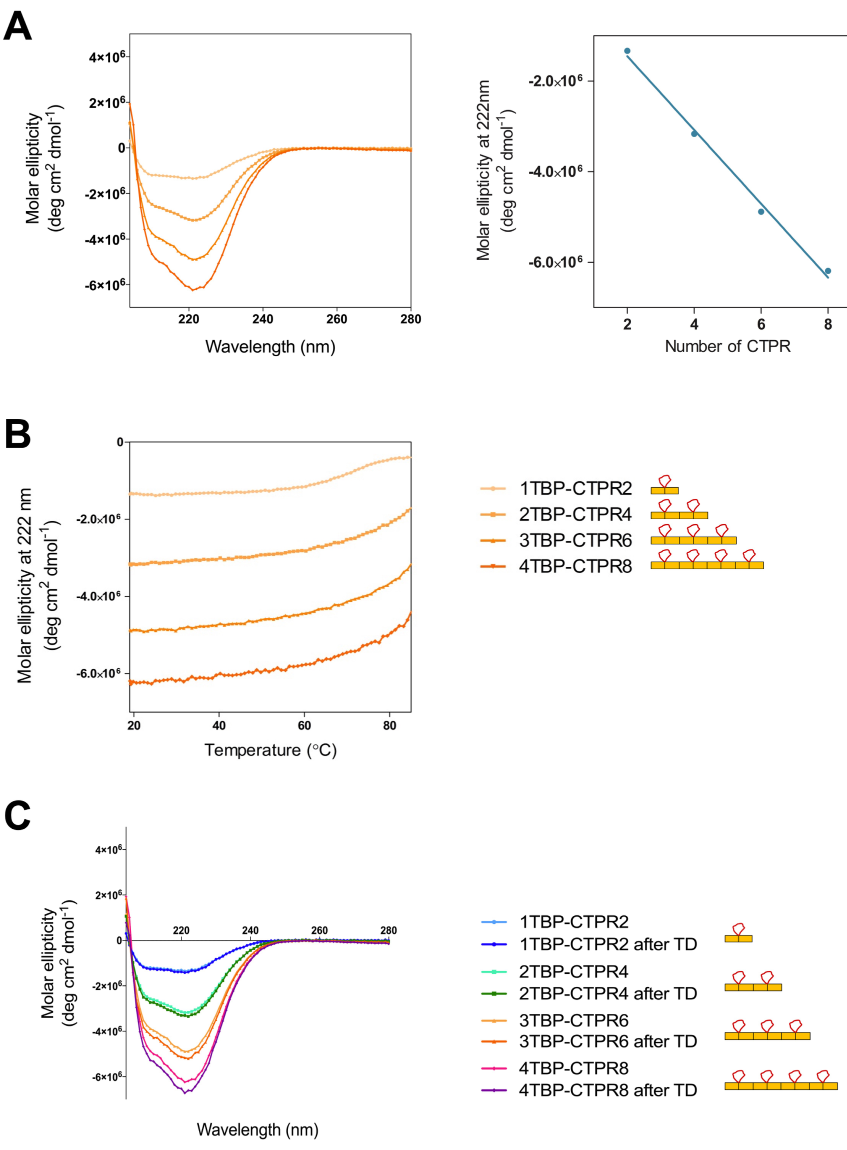

Figure S2. Chemical-induced denaturation of $_{n}$TBP-CTPR$_{2n}$ proteins.

Equilibrium denaturation curves of CTPR and $_{n}$TBP-CTPR$_{2n}$ proteins monitored by fluorescence. Protein concentration was 6-10 μM in 50 mM sodium phosphate pH 6.8, 150 mM NaCl at 25 ºC. The data are fitted to a two-state model.

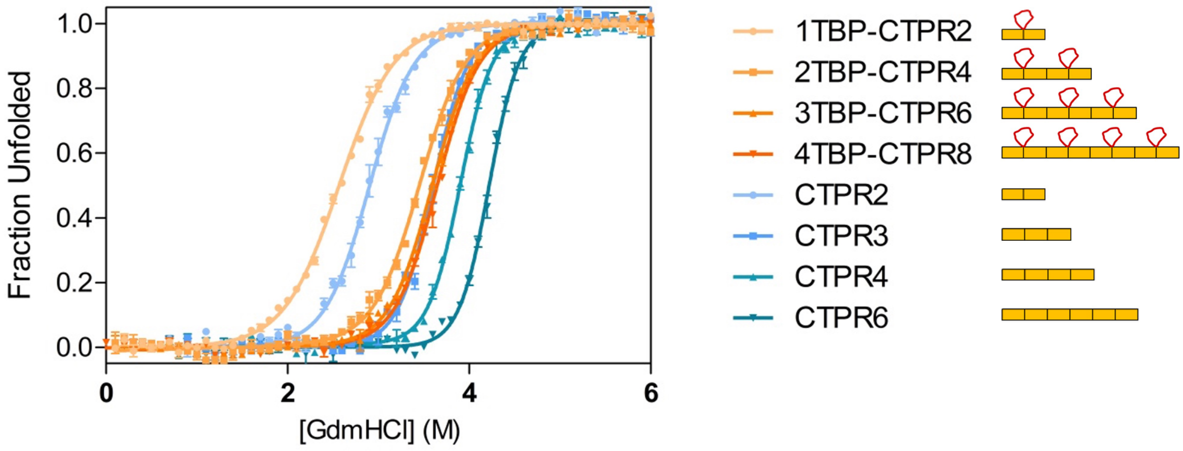

Table S2. Chemical denaturation parameters.

Values of $m_{\mathrm{D\text{-}N}}$, $D_{50\%}$ and ΔG_{D-N}^{H2O} obtained from a two-state fit of the equilibrium denaturation data. All measurements were performed in triplicate, and the errors listed are the SE of the mean.

Protein	$D_{50\%}$ (M)	$m_{\mathrm{D\text{-}N}}$ (kcal mol^{-1} M^{-1})	ΔG_{D-N}^{H2O} (kcal mol^{-1})
1TBP-CTPR2	2.57 ± 0.01	1.94 ± 0.05	-4.98 ± 0.13
2TBP-CTPR4	3.46 ± 0.01	2.33 ± 0.09	-8.06 ± 0.31
3TBP-CTPR6	3.60 ± 0.01	2.49 ± 0.06	-8.96 ± 0.22
4TBP-CTPR8	3.65 ± 0.01	2.55 ± 0.07	-9.31 ± 0.26
CTPR2	2.89 ± 0.02	2.42 ± 0.12	-6.99 ± 0.35
CTPR3	3.60 ± 0.01	3.06 ± 0.15	-11.02 ± 0.54
CTPR4	3.90 ± 0.01	3.37 ± 0.13	-13.14 ± 0.51
CTPR6	4.22 ± 0.01	3.54 ± 0.15	-14.94 ± 0.63

Table S3. ITC binding parameters.

Values of the changes in free energy (ΔG), enhalphy (ΔH) and entropy ($-T\Delta S$) for the interactions of the $_{n}$TBP-CTPR$_{2n}$ proteins with hTNKS2 ARC4 obtained by ITC. The values listed are from two independent experiments.

Protein	ΔG (kcal/mol)	ΔH (kcal/mol)	$-T\Delta S$ (kcal/mol)
1TBP-CTPR2	-6.60<br>-6.69	-15.81 ± 0.73<br>-24.87 ± 0.87	9.21<br>18.18
2TBP-CTPR4	-6.73<br>-6.47	-18.35 ± 0.26<br>-34.12 ± 0.32	11.62<br>27.65
3TBP-CTPR6	-7.18<br>-6.44	-12.39 ± 0.21<br>-35.85 ± 0.37	5.21<br>29.41
4TBP-CTPR8	-6.65<br>-6.59	-25.04 ± 0.21<br>-31.44 ± 0.48	18.39<br>24.85

Figure S3. ITC analysis of a control CTPR protein (CTPR6) lacking a TBP motif. TNKS2 ARC4 (200 µM) into CTPR6 (6.5 µM) at 25 ºC.

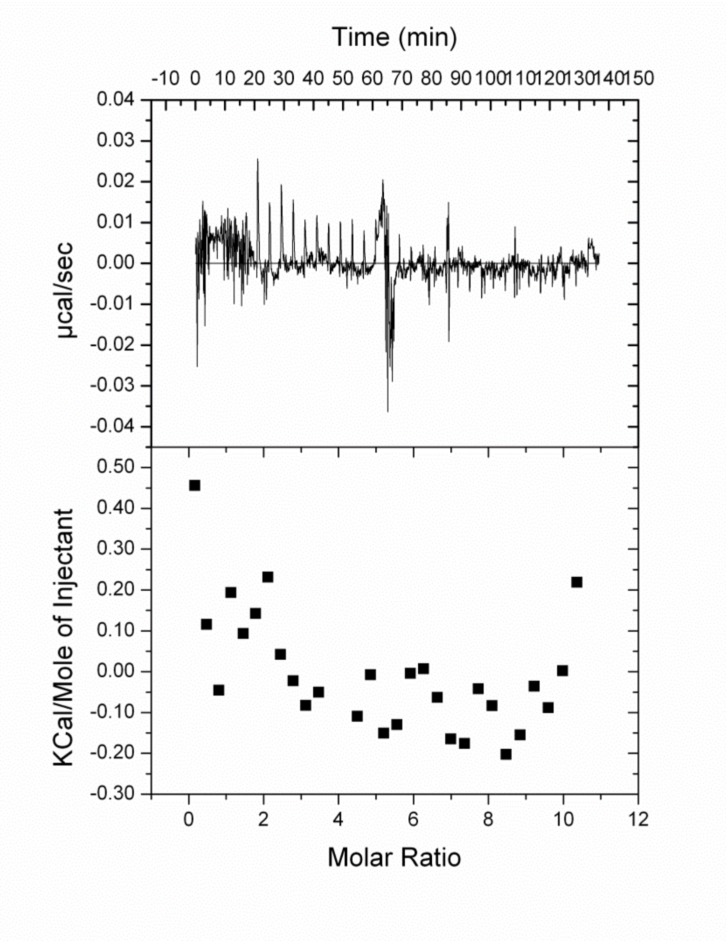

Figure S4. HiBiT-qIP assay analysis of interactions of transfected HiBiT-tagged hTNKS2 and HA-tagged 3TBP-CTPR6 or control bait constructs.

For each sample, the supernatant (SN), the final wash (Wash5) and the elution are shown. HiBiT-tagged hTNKS2 was pulled-down only when 3TBP-CTPR6 was used as bait. Comparable cellular content and loading was evaluated with α-Tubulin. α-Tubulin was found to bind non-specifically to the anti-HA resin, as it was detected in the eluted samples. The anti-HA blot confirms the presence of the desired protein bait constructs in the SN and in the eluted samples. The expression levels of 3TBP-CTPR6 and CTPR6 are consistent with those in Figure S10.

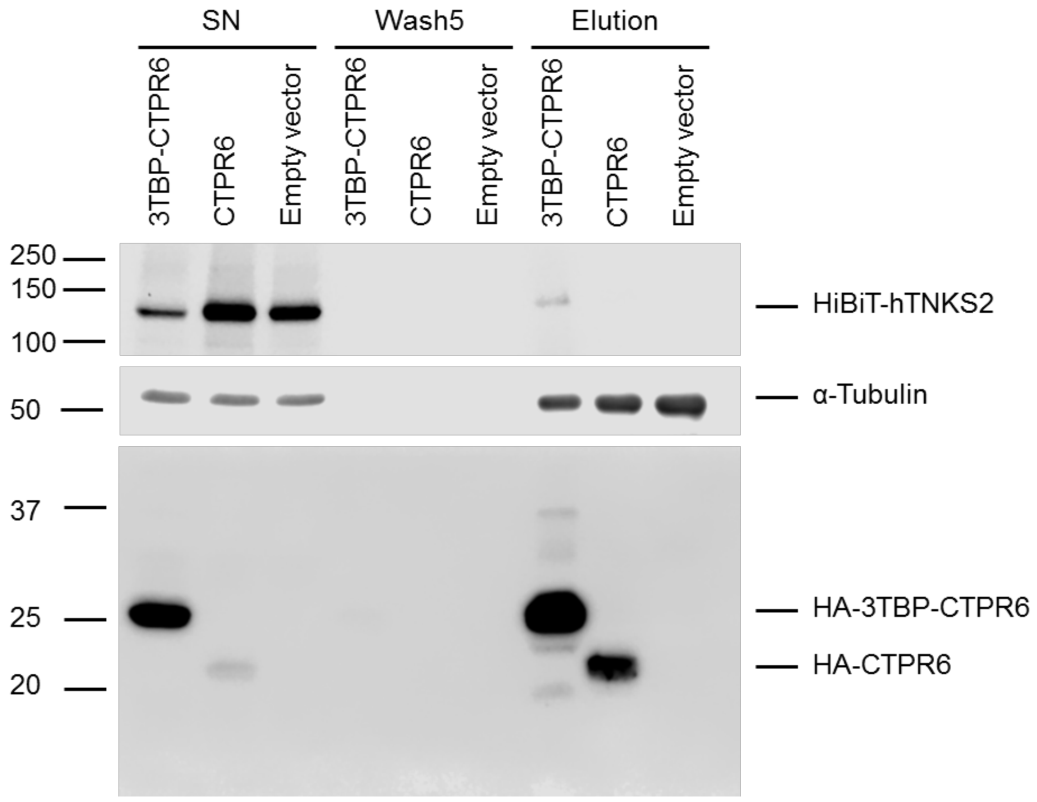

Figure S5. Design of trimeric ${}_{n}$TBP-CTPR$_{2n}$-foldon constructs.

Top: Sequence alignment of CTPR helix A (residues 1 to 15), the engineered CTPR helix A-foldon construct, and fibritin NCCF (residues 65 to 109). Rods represent α-helices, arrows represent β-strands, thin lines indicate unstructured regions.

Bottom: Native gel electrophoresis analysis of ${}_{n}$TBP-CTPR$_{2n}$ and ${}_{n}$TBP-CTPR$_{2n}$-foldon constructs.

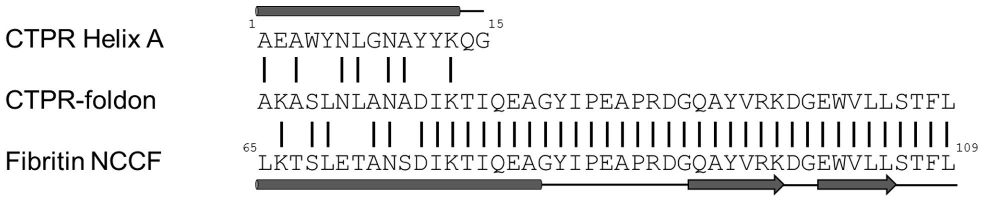

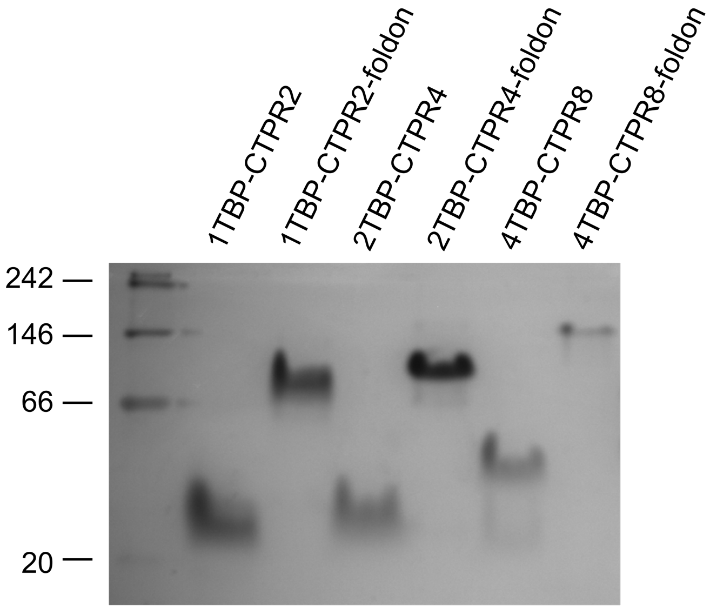

Figure S6. SEC-MALS analysis of 3TBP-CTPR6 and 3TBP-CTPR6-foldon proteins. The total protein concentration was 2.2 mg/mL for 3TBP-CTPR6 and 1.7 mg/mL for 3TBP-CTPR6-foldon. A schematic representation of the constructs used is provided on the right.

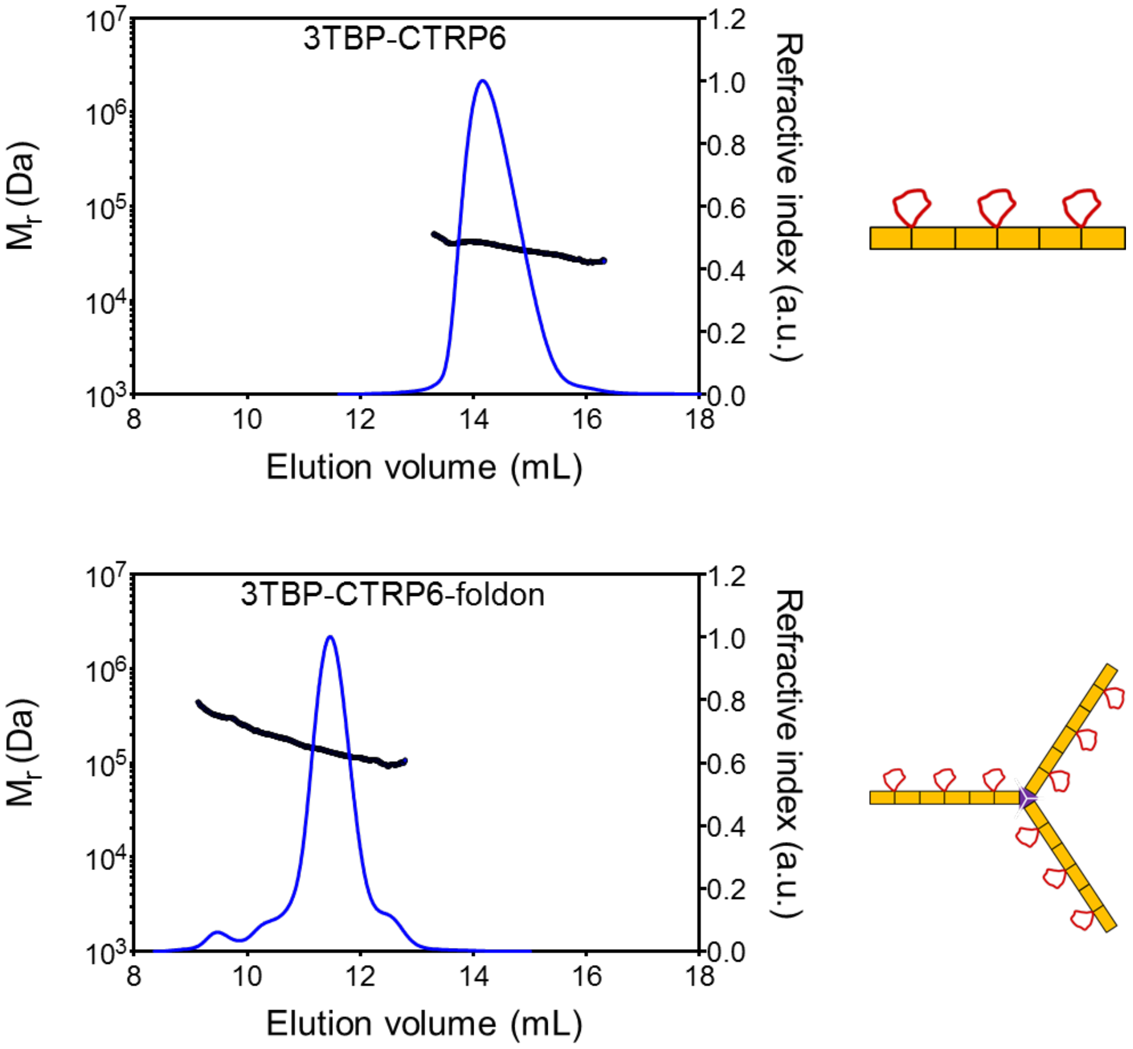

Figure S7. Co-precipitation experiments as a function of increasing CTPR protein concentration.

Samples were prepared by mixing a constant amount of hTNKS2 ARC1-3 (10 µM) with the indicated CTPR proteins at increasing concentrations (from 0 to 12.5 µM), in equal volumes. Therefore, the final protein concentration is halved. The trimeric 3TBP-CTPR6-foldon was prepared at a concentration three times lower the one of the linear 3TBP-CTPR6, for comparison purposes. After centrifugation, supernatants (S) and pellets (P) were separated and run on polyacrylamide gels. Gel band intensities were quantified and plotted on the left-hand side graphs. Knowing the initial protein concentration, the gel band intensities were converted into the corresponding protein concentration and plotted on the right-hand side graphs. Shown is a representative analysis of two independent experiments.

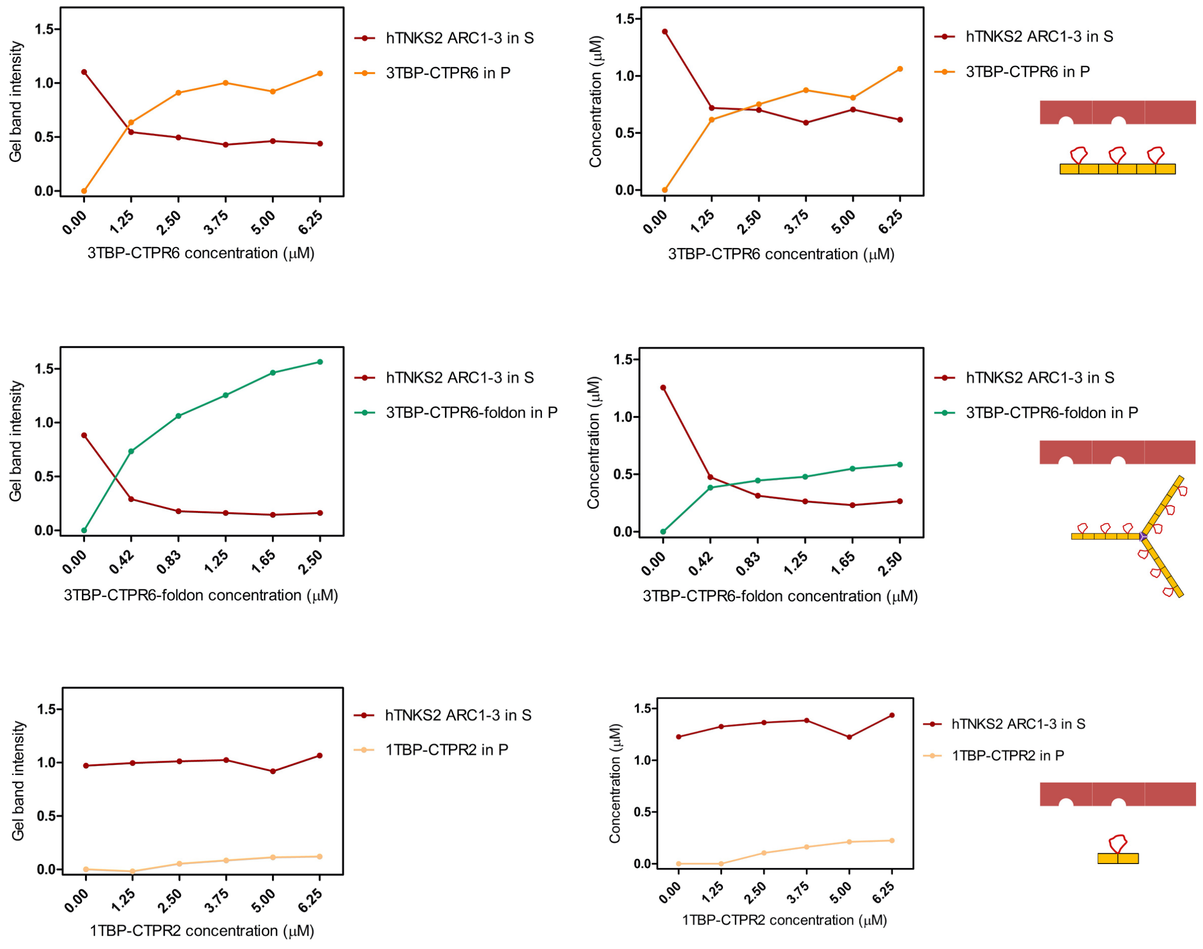

Table S4. Estimated stoichiometries of the assemblies formed between the multivalent $_{n}$TBP-CTPR$_{2n}$ proteins and the multivalent hTNKS2 ARC1-3 protein.

Multivalent 3TBP-CTPR6 and 3TBP-CTPR6-foldon, chosen as representatives of the linear and trimeric binding protein arrays, respectively, induced the formation of a macromolecular assemblies with the multivalent hTNKS2 protein. Equal volumes of supernatant (S) and pellet (P) for each sample were then loaded on a polyacrylamide gel and the resulting gel band intensity was measured. Using the starting concentrations of each proteins, it is possible to convert the gel band intensity into the molar concentration. The molar concentration of the protein constructs in the pellet provides information about the stoichiometry of the complexes. As shown in Figure 3, however, the hTNKS2 ARC1-3 protein showed some precipitation itself (see hTNKS2 ARC1-3-only samples S and P). Therefore, the molar concentration of hTNKS2 ARC1-3 in the pellet was corrected accordingly. The values obtained, as listed in Table S3, thus correspond to the amount of TBP-induced hTNKS2 ARC1-3 precipitation. The stoichiometry (ratio) is calculated by dividing the concentration of the CTPR protein by the concentration of the hTNKS2 ARC1-3 protein.

Initial 3TBP-CTPR6 (μM)	Initial hTNKS2 ARC1-3 (μM)	3TBP-CTPR6 in P (μM)	hTNKS2 ARC1-3 in P (μM)*	Ratio
0.00	5.00	0.00	0.00	-
1.25	5.00	0.62	0.67	0.92
2.50	5.00	0.75	0.69	1.09
3.75	5.00	0.88	0.80	1.10
5.00	5.00	0.81	0.68	1.18
6.25	5.00	1.06	0.77	1.37

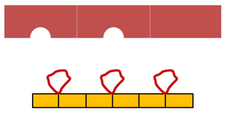

Initial 3TBP-CTPR6-foldon (μM)	Initial hTNKS2 ARC1-3 (μM)	3TBP-CTPR6-foldon in P (μM)	hTNKS2 ARC1-3 in P (μM)*	Ratio
0.00	5.00	0.00	0.00	-
0.40	5.00	0.38	0.78	0.49
0.83	5.00	0.45	0.94	0.47
1.25	5.00	0.48	0.99	0.48
1.65	5.00	0.55	1.02	0.54
2.50	5.00	0.58	0.99	0.59

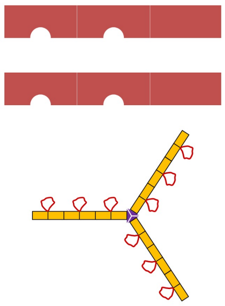

*Corrected for the self-precipitation of hTNKS2 ARC1-3.

Figure S8. Negative stain TEM.

hTNKS2 ARC1-3 was incubated with various single- and multivalent hTNKS-binding CTPR proteins and control CTPR proteins and imaged by negative stain TEM following a 1 h incubation. Proteins were mixed in equal volumes at the following concentrations: 5 μM 1TBP-CTPR2 (5 μM TBP loop concentration), 5 μM 3TBP-CTPR6 (15 μM TBP loop concentration), 1.7 μM 3TBP-CTPR6-foldon (15 μM TBP loop concentration), 5 μM CTPR6 and 5 μM hTNKS2 ARC1-3 (10 μM TBP-binding sites). Scale bars for all images are 500 nm. When hTNKS2 ARC1-3 was incubated with the multivalent 3TBP-CTPR6 or 3TBP-CTPR6-foldon, chosen as representative of the linear and trimeric arrays, respectively, large clustered structures, microns in size, were observed. Qualitatively, 3TBP-CTPR6 resulted in the formation of variable sized clusters throughout the TEM grid, ranging from small (1-2 μm clusters) to large networks many microns in size, while those formed by 3TBP-CTPR6-foldon appeared to be predominantly large, with smaller assemblies only occasionally visible. No other major differences could be identified between the two supramolecular assemblies. In contrast, samples of hTNKS2 ARC1-3 in combination with 1TBP-CTPR2, CTPR6 or alone showed some small amorphous aggregates.

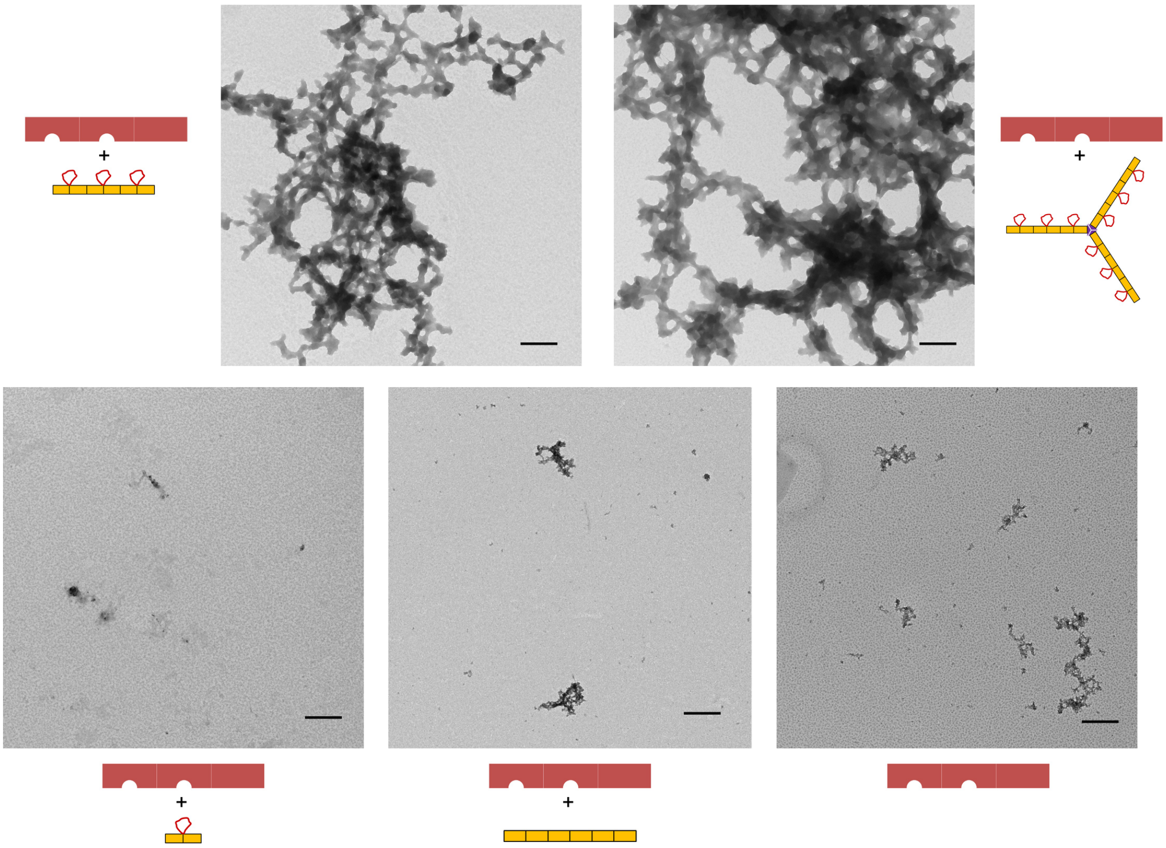

Figure S9. Fluorescence microscopy.

Fluorescence microscopy of HEK293T cells co-transfected with the e-GFP-tagged hTNKS2 ARC1 construct in combination with the mCherry-tagged CTPR constructs. The monovalent nature of the hTNKS2 ARC1 construct does not result in the formation of large macromolecular assemblies. Scale bars for all images are 10 µm.

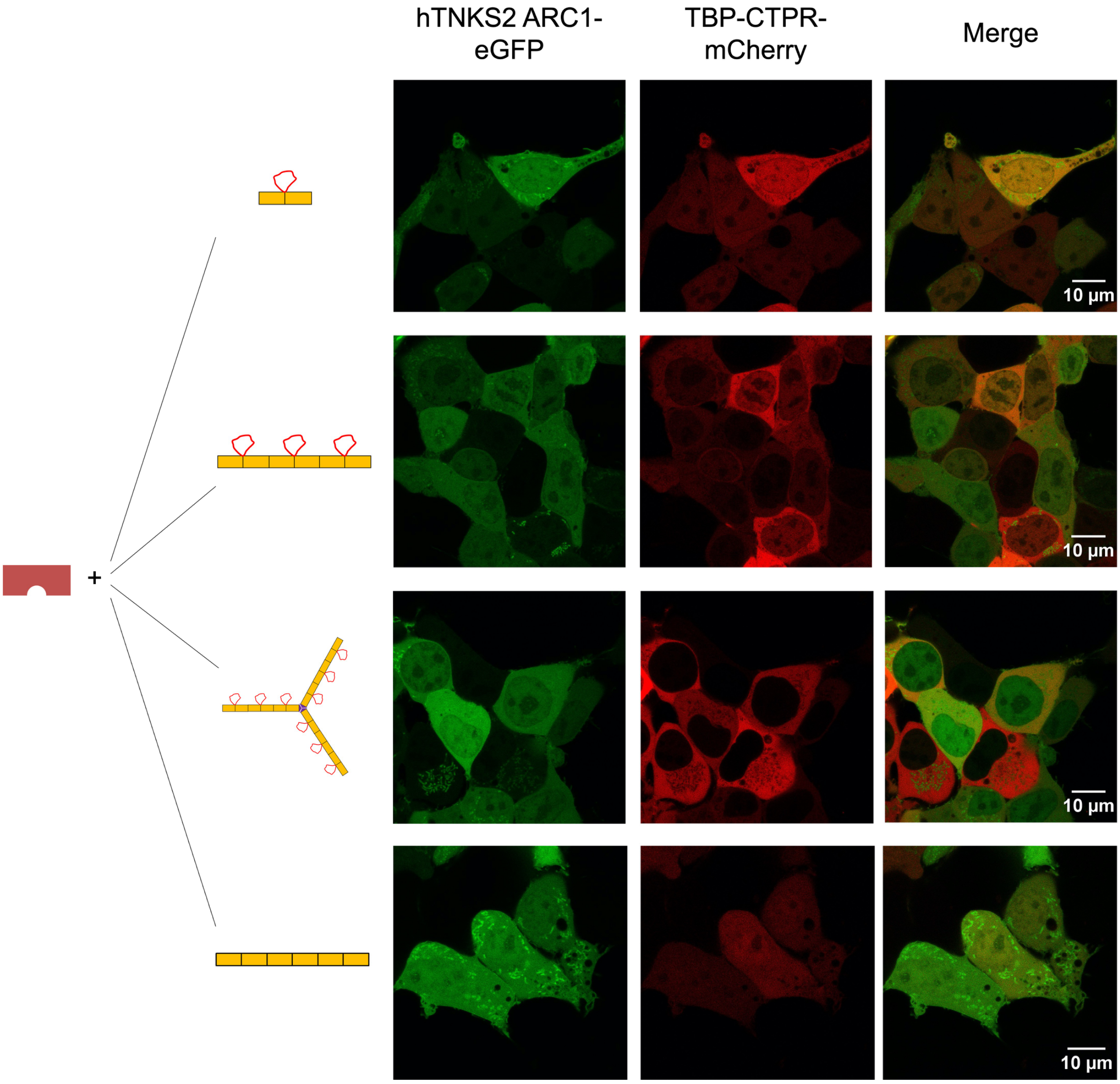

Figure S10. Expression levels of HiBiT-tagged $_n$TBP-CTPR$_{2n}$ proteins.

The levels of H-$_n$TBP-CTPR$_{2n}$ proteins, from the samples in Figure 6A, were measured by luminescence using HiBiT fusion proteins. Data were averaged and standard deviations were calculated from three independent sample measurements.

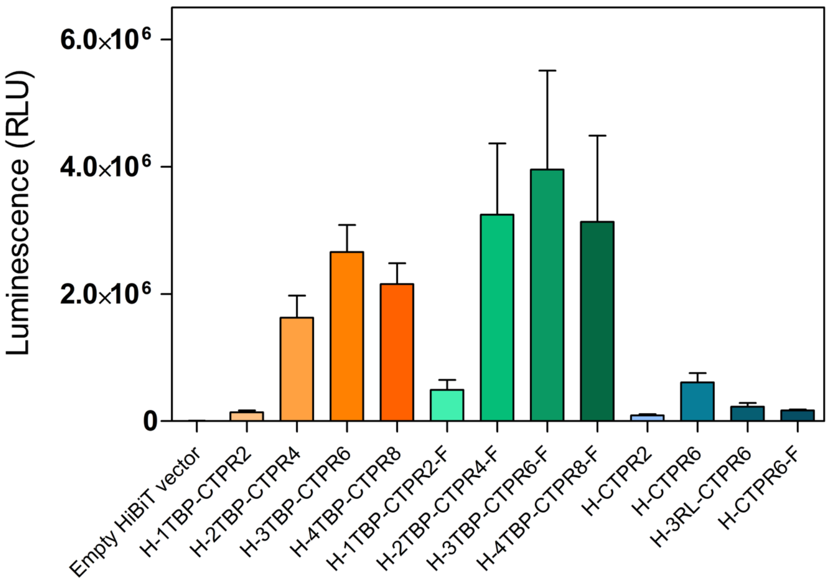

Figure S11. Expression levels of HiBiT-tagged $_{n}$TBP-CTPR$_{2n}$ proteins with MG132 treatment.

Fold increase in H-$_{n}$TBP-CTPR$_{2n}$ protein levels in the presence of the proteasome inhibitor MG132. Data were averaged and standard deviation was calculated from triplicate samples.

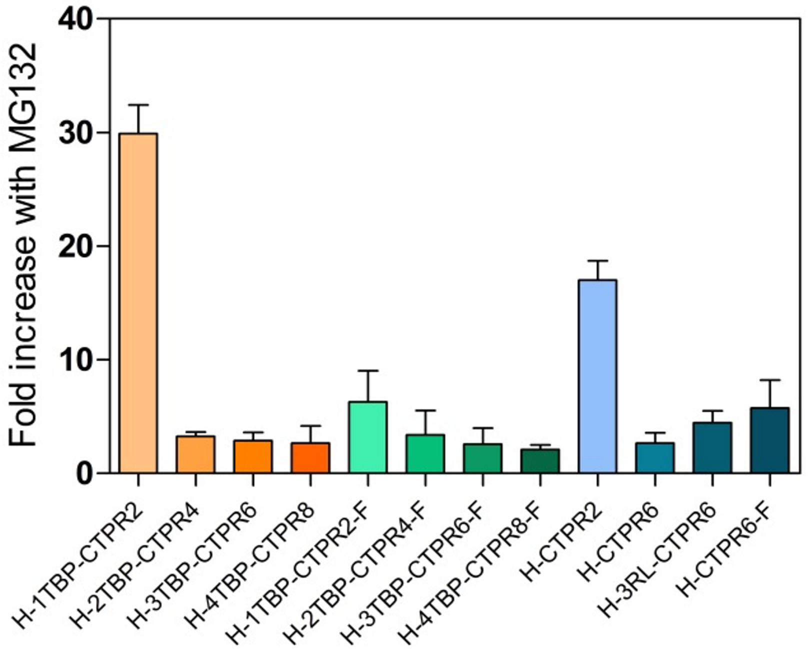

Figure S12. Cell viability assay.

Cell numbers in the presence or absence of MG132, following transfection with the indicated constructs.

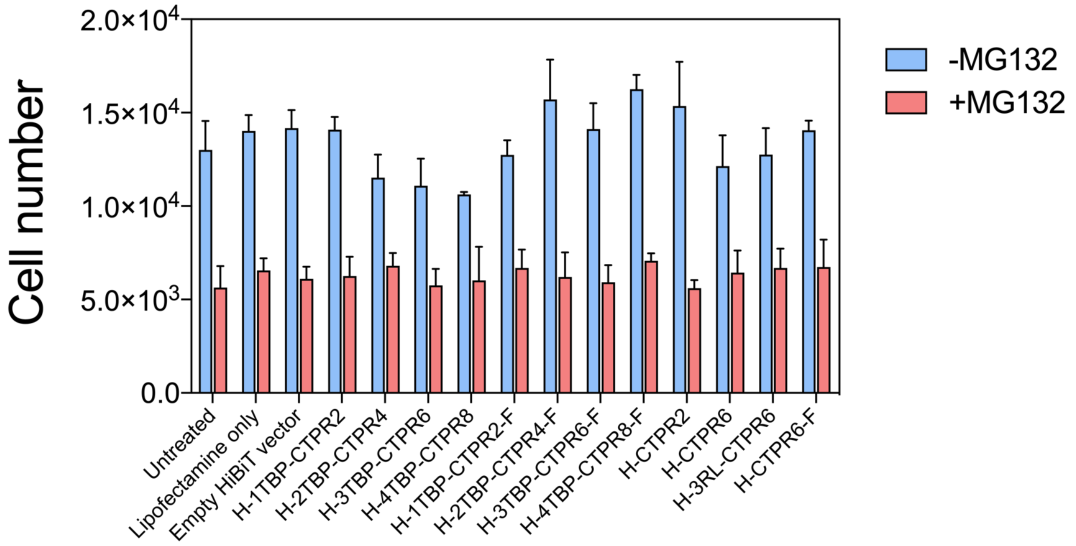

Figure S13. Characterisation of fusogenic liposomes (FL).

Surface charge of FL (a) and FL-3TBP-CTPR6 (b). Hydrodynamic size of FL (c) and FL-3TBP-CTPR6 (d).

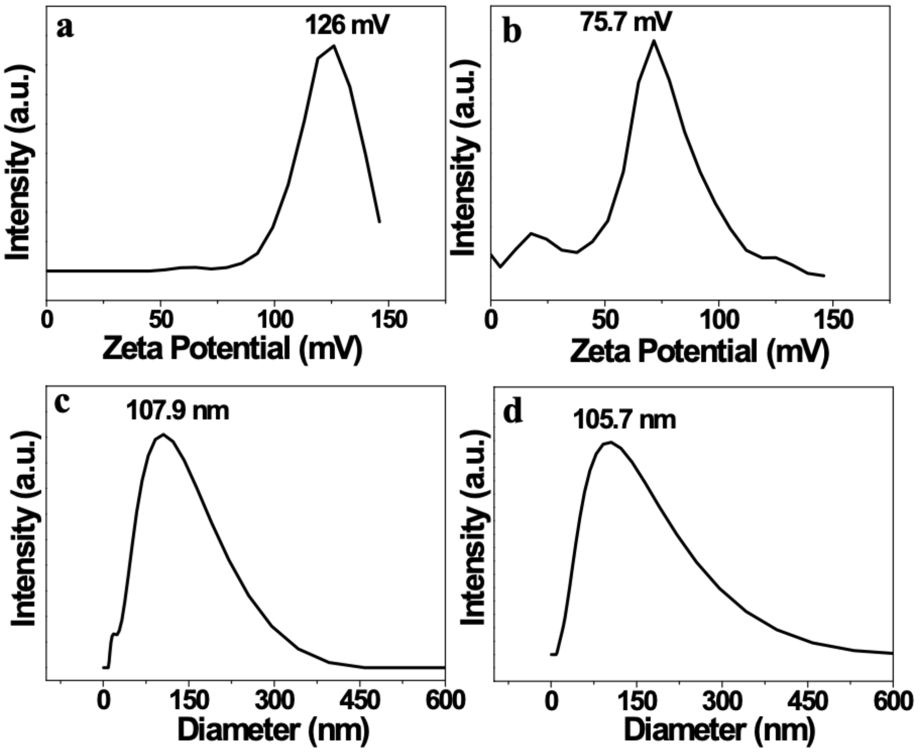

Figure S14. Cell viability assays of empty liposomes (FL, grey bars) and liposome-encapsulated 3TBP-CTPR6 protein (FL-3TBP-CTPR6; orange bars).

Untreated cells were taken as control for the experiment. Data were normalised relative to untreated cells set at 100%. Error bars were obtained from triplicate sample measurements from two independent experiments.

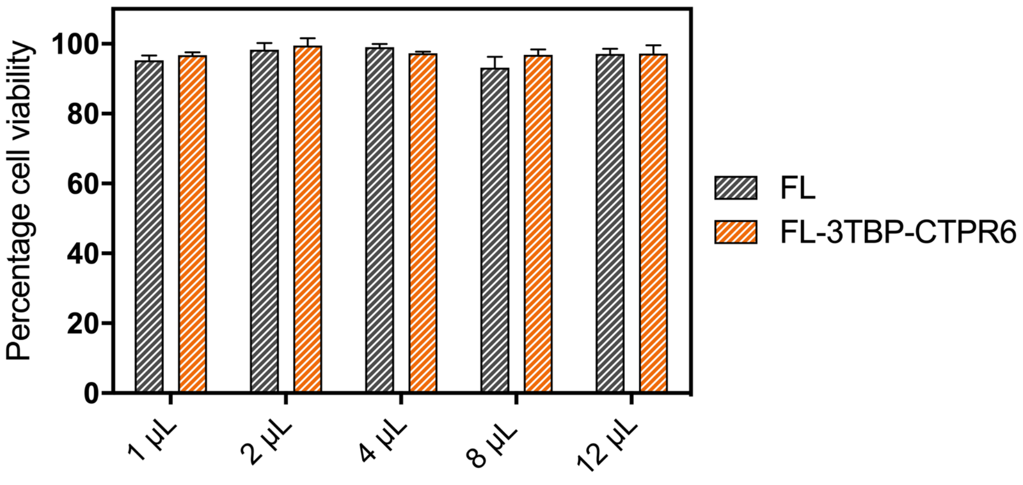

Figure S15. Confocal microscope images of HEK293T cells treated with liposome-encapsulated protein (FL-3TBP-CTPR6-RITC).

DiR (λ_{ex} = 633 nm, λ_{em} = 720-800 nm) stains the cellular membrane. 3TBP-CTPR6-RITC (λ_{ex} = 514 nm, λ_{em} = 530-650 nm) is distributed throughout cell cytoplasm. The merge of red and the green channel clearly shows that protein has been delivered inside the cells.

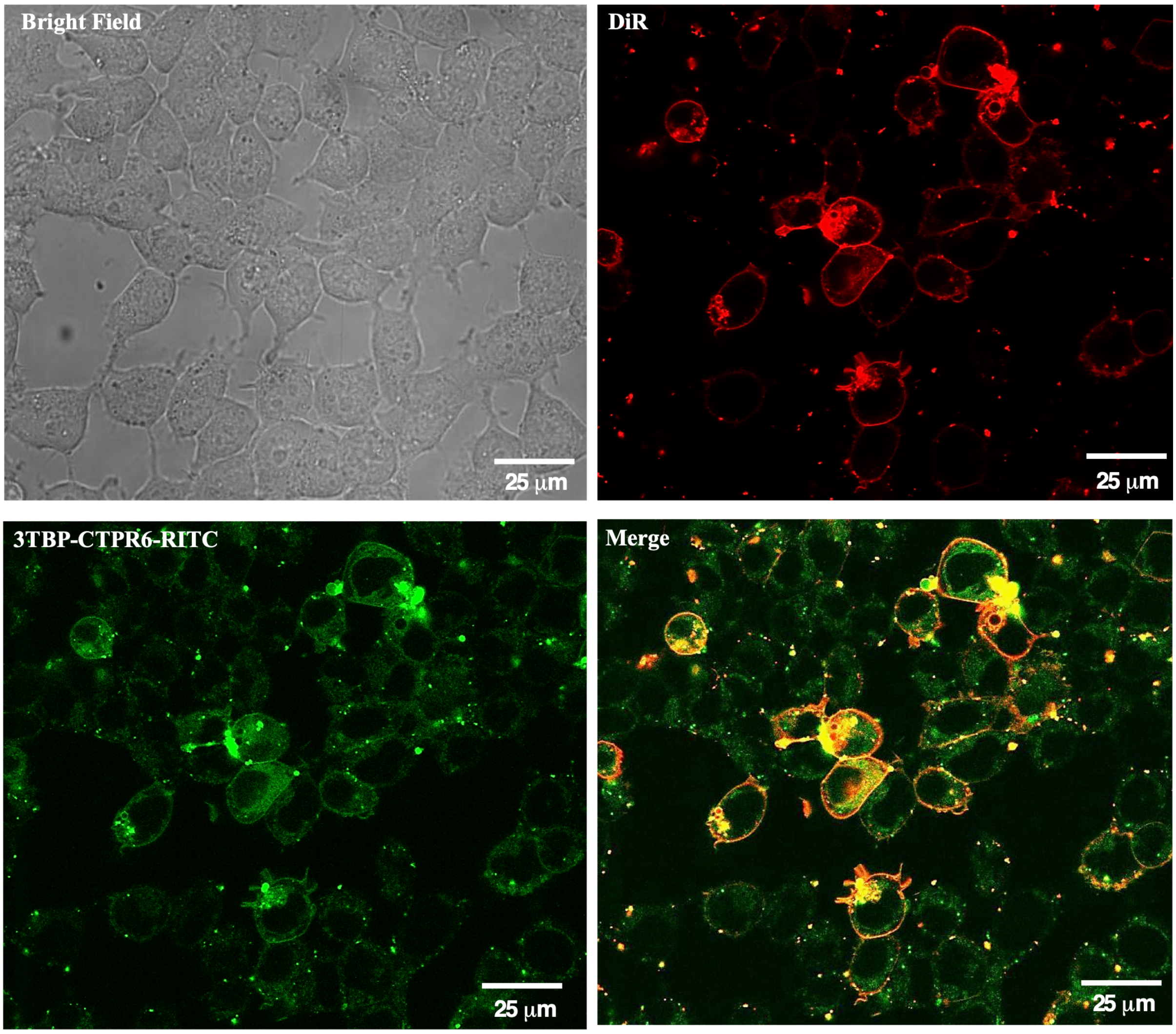

Figure S16. Confocal microscopy of HEK293T cells treated with empty fusogenic liposomes.

DiR (λ_{ex} = 633 nm, λ_{em} = 720-800 nm) stains the cellular membrane.

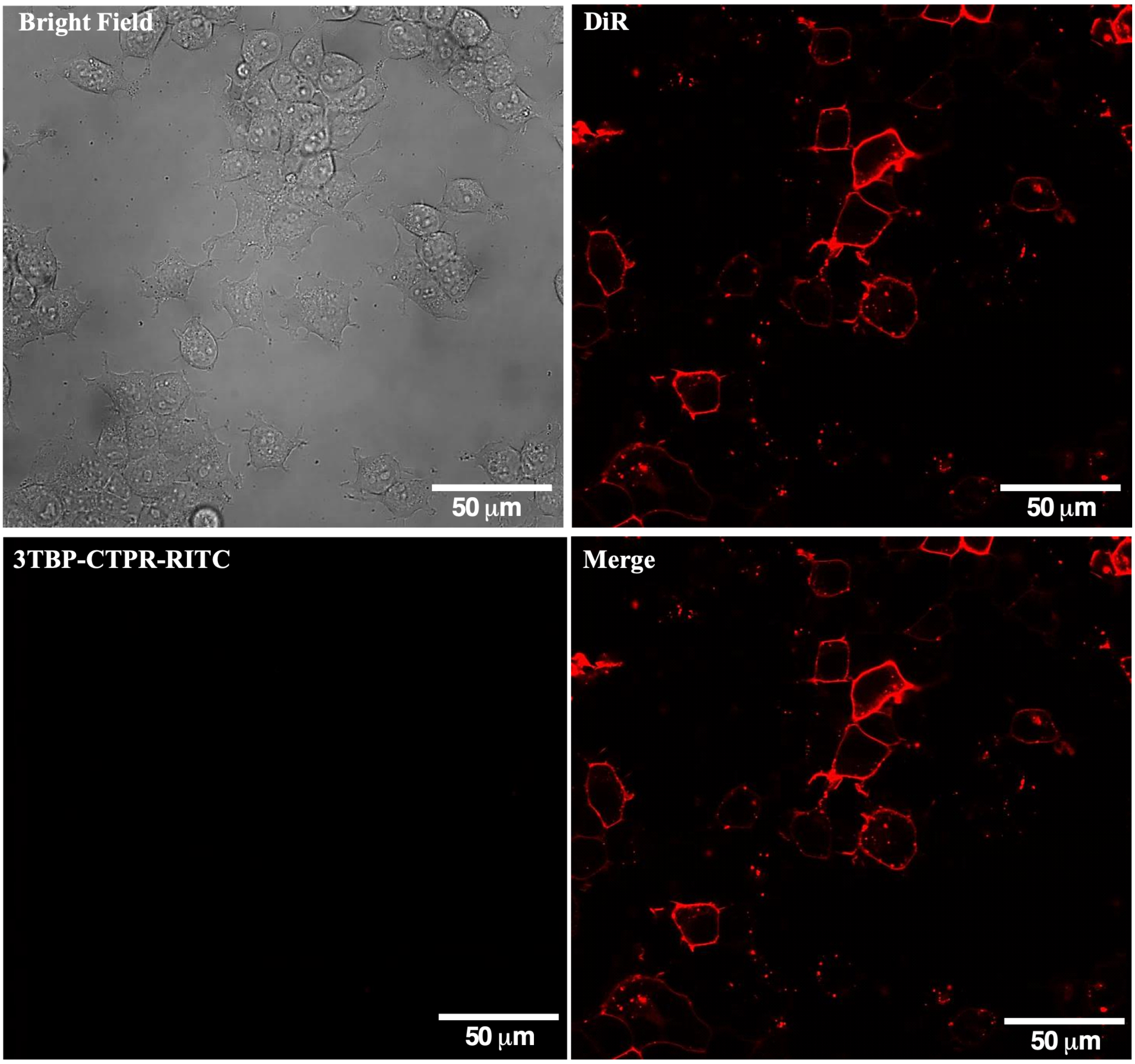

Figure S17. Effect of small molecule hTNKS inhibitors on Wnt signaling tested prophylactically.

For testing prophylactically, cells were treated with inhibitors mixed with Wnt-conditioned media and incubated for 16 hours. All the small molecule inhibitors were at a concentration of 1 μM in 0.5% DMSO. Data were normalised relative to the untreated control well, which was set at 100% (not shown in the graph). Error bars were determined from two independent sample measurements. The significance of the difference between data (***p≤0.001) was assessed using One-way ANOVA coupled with Dunnett's Multiple Correction test. hTNKS inhibitors were compared to DMSO treatment.

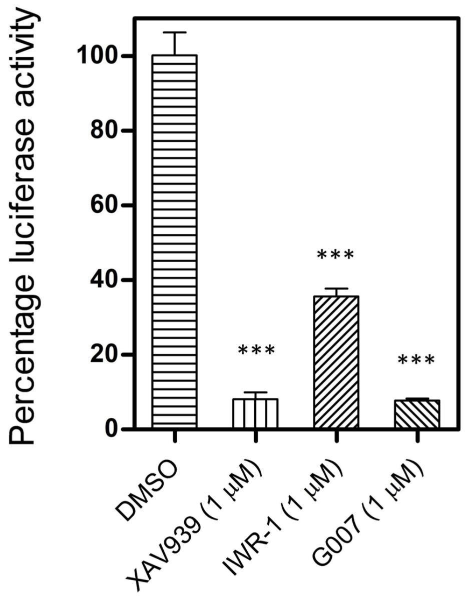

Supplement: Supplementary file 1 [file SC-012-D0SC03175E-s001.pdf]
